# Cross-frequency cortex–muscle interactions are abnormal in young people with dystonia

**DOI:** 10.1093/braincomms/fcae061

**Published:** 2024-02-26

**Authors:** Zhenghao Guo, Jean-Pierre Lin, Osvaldo Simeone, Kerry R Mills, Zoran Cvetkovic, Verity M McClelland

**Affiliations:** Department of Engineering, King's College London, London WC2R 2LS, UK; School of Biomedical Engineering, Dalian University of Technology, Dalian 116024, China; Children's Neuroscience, Evelina London Children's Hospital, Guy's & St Thomas' NHS Foundation Trust (GSTT), London SE1 7EH, UK; Department of Engineering, King's College London, London WC2R 2LS, UK; Department of Clinical Neuroscience, Institute of Psychiatry, Psychology and Neuroscience (IoPPN), King's College London, London SE5 9RX, UK; Department of Engineering, King's College London, London WC2R 2LS, UK; Children's Neuroscience, Evelina London Children's Hospital, Guy's & St Thomas' NHS Foundation Trust (GSTT), London SE1 7EH, UK; Department of Clinical Neuroscience, Institute of Psychiatry, Psychology and Neuroscience (IoPPN), King's College London, London SE5 9RX, UK

**Keywords:** cross-frequency coupling, sensorimotor processing, neuronal oscillations, spectral analysis, multi-scale wavelet transfer entropy

## Abstract

Sensory processing and sensorimotor integration are abnormal in dystonia, including impaired modulation of beta-corticomuscular coherence. However, cortex–muscle interactions in either direction are rarely described, with reports limited predominantly to investigation of linear coupling, using corticomuscular coherence or Granger causality. Information-theoretic tools such as transfer entropy detect both linear and non-linear interactions between processes. This observational case–control study applies transfer entropy to determine intra- and cross-frequency cortex–muscle coupling in young people with dystonia/dystonic cerebral palsy. Fifteen children with dystonia/dystonic cerebral palsy and 13 controls, aged 12–18 years, performed a grasp task with their dominant hand. Mechanical perturbations were provided by an electromechanical tapper. Bipolar scalp EEG over contralateral sensorimotor cortex and surface EMG over first dorsal interosseous were recorded. Multi-scale wavelet transfer entropy was applied to decompose signals into functional frequency bands of oscillatory activity and to quantify intra- and cross-frequency coupling between brain and muscle. Statistical significance against the null hypothesis of zero transfer entropy was established, setting individual 95% confidence thresholds. The proportion of individuals in each group showing significant transfer entropy for each frequency combination/direction was compared using Fisher's exact test, correcting for multiple comparisons. Intra-frequency transfer entropy was detected in all participants bidirectionally in the beta (16–32 Hz) range and in most participants from EEG to EMG in the alpha (8–16 Hz) range. Cross-frequency transfer entropy across multiple frequency bands was largely similar between groups, but a specific coupling from low-frequency EMG to beta EEG was significantly reduced in dystonia [*P* = 0.0061 (corrected)]. The demonstration of bidirectional cortex–muscle communication in dystonia emphasizes the value of transfer entropy for exploring neural communications in neurological disorders. The novel finding of diminished coupling from low-frequency EMG to beta EEG in dystonia suggests impaired cortical feedback of proprioceptive information with a specific frequency signature that could be relevant to the origin of the excessive low-frequency drive to muscle.

## Teaser

Guo *et al.* investigate cortex–muscle interactions in young people with dystonia. Using the information theoretical technique of transfer entropy, they demonstrate impaired cortical feedback of proprioceptive information with a specific frequency signature in dystonia, which could potentially serve as a biomarker.

## Graphical Abstract

**Graphical Abstract:**
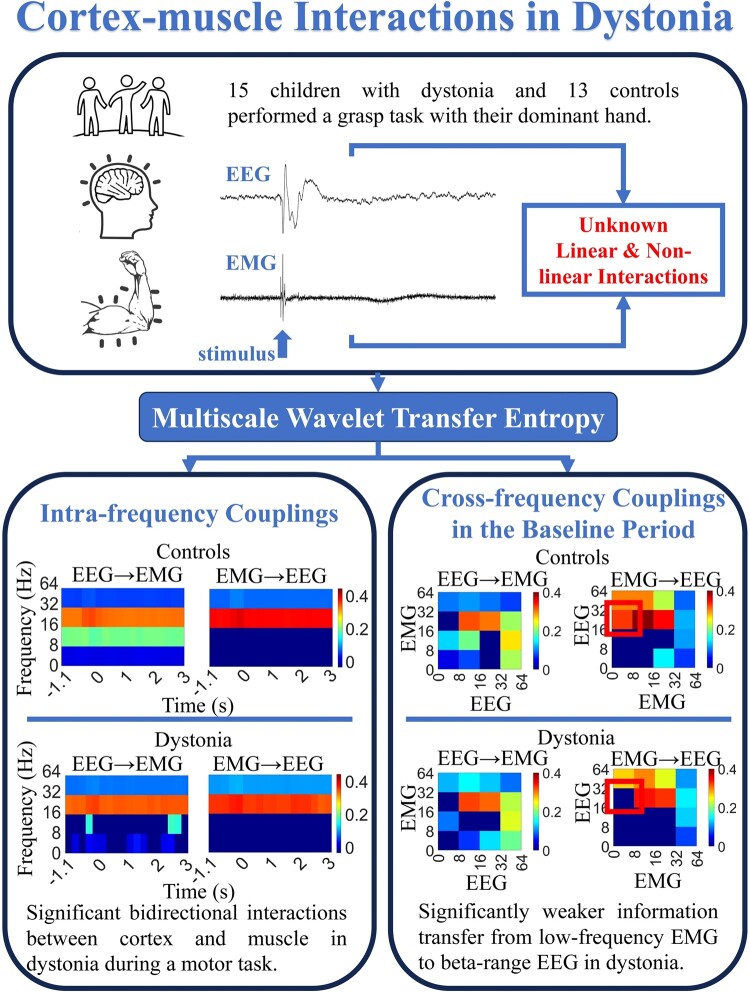

## Introduction

Dystonia is a severe movement disorder with no cure, characterized clinically by involuntary, sustained, or intermittent, muscle contractions causing abnormal, often repetitive, movements, postures or both.^1^ Dystonia can be considered as a syndrome or motor symptom that can arise from many different aetiologies,^1,2^ may involve brain lesions in many different anatomical sites (e.g. basal ganglia, thalamus, sensorimotor cortex and cerebellum)^3^ or may be associated with no overt brain anatomical lesion on structural cranial MRI.^3,4^ Multiple pathophysiological mechanisms have been implicated,^5,6,7-9,10-15,16-19^ all accommodated by the network model of dystonia, which proposes that dystonia arises from dysfunction within the basal ganglia, thalamus, sensorimotor cortex and cerebellum and/or their interconnections.^3,6,20^ This concept is further supported by a recent fluorodeoxyglucose positron emission tomography study that revealed divergent patterns of abnormal brain glucose metabolism across different brain regions in 10 aetiologically distinct dystonia groups. For example, pallidal and/or putaminal hypometabolism were seen as common findings in most groups, parietal and frontal hypermetabolism were prominent in dystonic cerebral palsy, but PANK2 neurodegeneration with brain iron accumulation was characterized by parietal hypermetabolism, cerebellar hypometabolism and preserved putaminal–pallidal metabolism,^21^ demonstrating how the network above may be disrupted differentially between dystonia aetiologies.

One of the striking features of dystonia is pathologically enhanced low-frequency neuronal oscillations within this network^22-25^ that are coherent with dystonic EMG.^22,23^ Another feature common across many genetic, idiopathic and acquired aetiologies is an abnormally enhanced low-frequency (4–12 Hz) drive to muscles, as revealed by studies of intermuscular coherence in cervical dystonia, DYT1 dystonia and DYT11 dystonia^26-31^ as well as in acquired dystonia.^31^ This exaggerated low-frequency intermuscular coherence correlates with dystonia severity^30,31^ suggesting that it is of clinical importance. Beta-corticomuscular coherence (CMC) is also abnormal in dystonia, with very low levels of beta-CMC seen in individuals with genetic/idiopathic dystonias.^26,31^ Interestingly, the genetic/idiopathic dystonia group also showed a lack of modulation of beta-CMC in response to a sensory stimulus, suggesting an abnormality of sensorimotor integration,^31^ whereas the same study found that most patients with ‘acquired’ dystonia modulated their CMC in a similar pattern to controls, suggesting a possible divergence of some sensorimotor processing parameters between genetic/idiopathic and acquired dystonias.

Standard CMC analysis reveals interactions between cortex and muscle,^32^ while directed coherence analysis/Granger causality can add further information on the direction of the interaction.^33-36^ However, both techniques are limited by only being able to detect linear interactions and therefore lack sensitivity.^37^ A linear interaction is one in which one process propagates to another via a single path or multiple paths, each of which may introduce attenuation and delay, hence any change in input amplitude or phase leads to the same change in output, and always within the same frequency band. All other interactions (within or across frequency bands) are non-linear. The nervous system is highly complex and exhibits non-linear processes and behaviours at multiple levels ranging from single neurons to entire networks.^38^ Given that synaptic function is not linear, there is likely to be greater non-linearity in polysynaptic than in monosynaptic pathways.^39^ Thus the somatosensory pathway, which contains a greater number of synapses than the corticospinal motor pathway, is less well described by linear models. Activity in multi-synaptic motor pathways such as the reticulospinal tract will also be poorly detected. Methods that allow detection of both linear and non-linear neural interactions are therefore gaining prominence as important tools for providing a better understanding of sensorimotor systems in health and disease.^38,40,41^ In particular, for dystonia, where there is growing evidence of somatosensory system disruption and in which the network model incorporates multiple polysynaptic pathways, it is likely that techniques with capacity to delineate a broader range of interactions, including linear and non-linear communication between nodes of the network, will be informative.

Information-theoretic tools such as transfer entropy (TE) are capable of detecting and quantifying both linear and non-linear interactions between processes.^42-44^ The recently developed technique of multi-scale wavelet transfer entropy (MWTE) applies TE in conjunction with the wavelet transform to decompose the signals into distinct frequency bands, and uses multi-scale embedding parameters to detect and quantify intra- and cross-frequency band coupling between cortex and muscle at different time scales.^37^ We recently applied this technique to neurophysiological data from healthy adults acquired during a controlled motor task, and revealed significant interactions between cortex and muscle in both directions in all participants, even in those individuals who did not demonstrate significant coupling with either CMC or directed coherence/Granger causality analysis.^37^ This is likely to indicate the presence of non-linearities in the bidirectional communications between cortex and muscle.

In view of the reported reduction in cortex–muscle communication in young people with dystonia, as measured with traditional CMC analysis,^26,31^ we investigated this further aiming to explore non-linear interactions both within and across frequency bands. We applied the MWTE methodology to the data collected in the McClelland *et al.*^31^ study to test the hypothesis that individuals with dystonia will demonstrate significant bidirectional cortex–muscle interactions. As a secondary analysis, we also explored cross-frequency coupling (CFC) in individuals with dystonia and tested the hypothesis that patterns of communication across frequency bands would differ between healthy controls and individuals with dystonia.

## Materials and methods

The analyses were performed on data acquired in a previously published study of CMC in children and young people with dystonia.^31^ Detailed description of the experimental procedures is found in that paper, but summarized here.

### Ethical approval

Ethical approval was obtained from the London-Fulham National Research Ethics Committee, London, UK (12/LO/0925). Informed written consent was obtained from the participant or, if under 16 years old, from parents with assent from the child. The studies were conducted in accordance with the Declaration of Helsinki.

### Subjects and experimental arrangement

The participants were 15 children with dystonia (10 acquired, five idiopathic/genetic, nine female), recruited from the Complex Motor Disorders Service at Evelina London Children's Hospital from 2014–16, and 13 typically developing children (seven female). Age range was 12–18 years. Details are given in Table 1, adapted from the original report.^31^ The diagnosis and classification of dystonia were confirmed by a consultant paediatric neurologist with specialist expertise in movement disorders (J.-P.L.). One further participant from the original study, with acquired dystonia, was excluded from the current analysis due to a large number of data epochs being contaminated by excessive movement artefact.

**Table 1:** Clinical details

Case no.	Group	Age at study	Phenotype (Classification Axis 1)^a^	Diagnosis (Classification Axis 2)^a^	Location of MRI abnormalities	Clinical scales		
						GMFCS	MACS	BFMDRS-d/m
P1	Isolated genetic/idiopathic	12	Generalized partially dopa-responsive dystonia	Idiopathic	Normal	3	3	8/52
P2	Isolated genetic/idiopathic	13	Generalized dystonia	Genetic—DYT1 mutation	Normal	1	2	7/11
P4	Isolated genetic/idiopathic	18	Generalized dystonic choreoathetosis with possible myoclonic elements. Whispering dysphonia	Genetic—KMT2B mutation	BG	2	3	17/72
P6	Isolated genetic/idiopathic	12	Generalized dystonia-dyskinesia	Idiopathic—family history of dopa-responsive dystonia	Normal	1	3	12/39.5
P10	Isolated genetic/idiopathic	17	Generalized dystonia with myoclonus	Genetic—TITF1 mutation	Normal	1	2	9/24.5
P3	Acquired	15	Generalized dystonia-dyskinesia	Cerebral palsy secondary to Perinatal HIE	WM	1	2	10/24
P5	Acquired	15	Generalized dystonia-dyskinesia	Cerebral palsy secondary to perinatal HIE	Normal	2	2	8/60
P7	Acquired	18	Generalized dystonia onset age 13	Unknown. Mild white matter changes on MRI	WM	1	1	7/36.5
P8	Acquired	15	Generalized asymmetric dystonia Asymmetry right > left	Presumed perinatal injury	BG, WM, cortex	2	3	N/A
P9	Acquired	17	Generalized asymmetric dystonia Asymmetry right > left	Perinatal arrested hydrocephalus	BG, WM	2	2	7/54.5
P11	Acquired	17	Generalized asymmetric dystonia Asymmetry left > right	Right middle cerebral artery infarct	BG, WM, cortex	1	2	N/A
P12	Acquired	14	Generalized dystonia + choreoathetosis	Glutaric aciduria with symmetrical gliosis of putamina bilaterally	BG	2	3	N/A
P13	Acquired	18	Early onset generalized dystonia from 11 months. Severe expressive language difficulties	Unknown	BG	5	4	N/A
P14	Acquired	13	Generalized dystonia + athetosis	Cerebral palsy secondary to perinatal HIE	BG	2	2	14/51
P15	Acquired	14	Generalized dystonia + athetosis	Cerebral palsy secondary to perinatal HIE	BG, WM	2	2	12/53

MRI, magnetic resonance imaging; BG, basal ganglia; WM, white matter; GMFCS, Gross Motor Function Classification System score; MACS, Manual Ability Classification System score; BFMDRS, Burke Fahn Marsden Classification System—motor score; HIE, hypoxic ischaemic encephalopathy; N/A, not available.

^a^Classification is given according to Albanese *et al*.^1^

Subjects performed a simple motor task with their dominant hand (13 controls and 11 children with dystonia were right hand dominant by self-report), which involved grasping a 15 cm plastic ruler in a key grip between the thumb and index finger. Mechanical perturbations to the task were provided by an electromechanical tapper driven by a power amplifier (Ling Dynamic Systems Limited) as detailed previously.^45^ The tapper provided pulses of lateral displacement (1 mm at a velocity of 0.2 m/s), giving the participant the sensation that their grip on the ruler may be lost. The perturbation had a rise-time of 5 ms and duration of 20 ms. The stimuli were delivered at pseudorandom intervals between 5.6 and 8.4 s (mean 7 s). Stimulus amplitude was constant throughout the experiment and between participants. Stimuli were delivered in blocks of 10–25, depending on the participant's ability to maintain performance, up to 200 epochs in total. Brief rest periods were given between blocks. A maximum voluntary contraction (MVC) was also recorded for each patient, measuring the highest of three attempts.

### EEG and EMG recording

EMG was recorded using adhesive electrodes placed in a belly-tendon montage over dominant first dorsal interosseous (FDI). Bipolar EEG was recorded from scalp overlying the contralateral hand area of motor cortex, one electrode positioned 5 cm lateral to the vertex along the interaural line and the other 2.5 cm anterior to it.^45-47^ Scalp electrodes were applied using conductive paste, and impedance reduced below 5 kOhm.

### Data processing

EEG and EMG signals were sampled at 1024 Hz, amplified and band-pass filtered (0.5–100 Hz for EEG; 5–500 Hz for EMG). The data were segmented into epochs lasting 5 s, with the stimulus delivered 1.1 second after the start of the data collection period. Raw data were reviewed offline by visual inspection, and epochs of data containing movement or blink artefacts were rejected. A notch filter was applied to remove the 50 Hz mains interference. Pre-processed data were then analysed using the MWTE methodology, which is described in detail in Guo *et al*.^37^ and summarized below. EMG data were not rectified, in accordance with the previously published methodology.^37^

### Transfer entropy

TE is a generalized time-domain measure for quantifying directional and dynamical information flows between random processes.^43^ Given two time series of interest $\left\{x_{t}\right\}$ and $\left\{y_{t}\right\}$, and their interaction delay u≥0, TE measures the amount of information provided by past samples of the source process


(1a) $$x_{t-1}^{\tau_{x},d_{x}}=(x_{t-(d_{x}-1)\tau_{x}-1},x_{t-(d_{x}-2)\tau_{x}-1},\ldots ,x_{t-1}),$$

about the next state of the target process $y_{t}$ given its past samples


(1b) $$y_{t+u-1}^{\tau_{y},d_{y}}=(y_{t-(d_{y}-1)\tau_{y}+u-1},y_{t-(d_{y}-2)\tau_{y}+u-1},\ldots ,y_{t+u-1}),$$

where $\tau_{x}$ and $\tau_{y}$ are known as embedding delays, and $d_{x}$ and $d_{y}$ are known as embedding dimensions for the time series $\left\{x_{t}\right\}$ and $\left\{y_{t}\right\}$, respectively. TE is formally defined as^44^


(2) $$\begin{array}{rl} TE_{x\to y}(\tau_{x},d_{x},\tau_{y},d_{y},u) & =I(x_{t-1}^{\tau_{x},d_{x}};y_{t+u}|y_{t+u-1}^{\tau_{y},d_{y}})\\ & =E\left[\log\left(\frac{\;p(y_{t+u}|x_{t-1}^{\tau_{x},d_{x}},y_{t+u-1}^{\tau_{y},d_{y}})}{\;p(y_{t+u}|y_{t+u-1}^{\tau_{y},d_{y}})}\right)\right], \end{array}$$

where I(x;y|z) represents the conditional mutual information of jointly distributed random variables (x,y,z), and the expectation is taken over their joint probability distribution.

### Multi-scale wavelet transfer entropy

In order to identify information transfer within and across functional frequency bands of neural oscillations at different time scales, we further considered the MWTE approach.^37^ The methodology relies on a dyadic stationary wavelet transform (SWT) that approximately decomposes the neurophysiological signals into functional bands. Our focus here lies in the analysis of neural oscillations within and across these functional frequency bands, due to their physiological relevance in sensorimotor control. Processing a signal $\left\{x_{t}\right\}$ with SWT at level *J* produces J+1 sub-band components $\{x_{\;j,t}\},\;j=1,\ldots ,J+1$


(3) $$x_{\;j,t}=\{\begin{array}{c} h_{J,0}\;*\;x_{t}\;,\;j=J+1\\ h_{\;j,1}\;*\;x_{t}\;,\;j=1,\ldots ,J \end{array},$$

where $h_{\;j,0}$ and $h_{\;j,1}$ are the underlying discrete-time filters at level *j*.^48^ Based on the sub-band components $x_{\;j,t}$, the multi-scale is implemented by setting embedding delays *τ* in (1) across the wavelet transform scales as


(4a) $$\tau_{j}=s\tau_{j}^{\left(0\right)},$$

(4b) $$\tau_{\;j+1}^{\left(0\right)}=2\tau_{j}^{\left(0\right)},$$

where *j* is the scale index of the wavelet transform, $\tau_{j}^{\left(0\right)}$ is the minimal embedding delay at scale *j* and *s* is a positive integer that controls the timescale of the TE analysis. Thus, the delay embedding vectors (1) become


(5a) $$x_{\;j_{x},t-1}^{\tau_{\;j_{x}},d_{x}}=(x_{\;j_{x},t-(d_{x}-1)\tau_{\;j_{x}}-1},\ldots ,x_{\;j_{x},t-1}),$$

(5b) $$y_{\;j_{y},t+u-1}^{\tau_{\;j_{y}},d_{y}}=(y_{\;j_{y},t-(d_{y}-1)\tau_{\;j_{y}}+u-1},\ldots ,y_{\;j_{y},t+u-1}),$$

where $x_{\;j_{x},t}$ denotes the sample at time instant *t* of the sub-band component $\left\{x_{\;j_{x},t}\right\}$ of $\left\{x_{t}\right\}$. Finally, the MWTE is defined as^37^


(6) $$\mathrm{MWT}E_{x_{\;j_{x}}\to y_{\;j_{y}}}(\tau_{\;j_{x}},d_{x},\tau_{\;j_{y}},d_{y},u)=I(x_{\;j_{x},t-1}^{\tau_{\;j_{x}},d_{x}};y_{\;j_{y},t+u}|y_{\;j_{y},t+u-1}^{\tau_{\;j_{y}},d_{y}}).$$

In this study, the wavelet transform was performed at J=6 scales. Since the signals were recorded using 1024 Hz sampling, the sub-band components $x_{4,t}$, $x_{5,t}$, $x_{6,t}$ and $x_{7,t}$ approximately represent (32–64) Hz—low gamma, (16–32) Hz—beta, (8–16) Hz—alpha, and (0–8) Hz—the combination of theta and delta bands. The decision not to separate delta and theta oscillations is governed by the data acquisition process during which most of the 0–4 Hz EMG content was filtered out, as is common in motor neurophysiology studies, to minimize movement artefact. We employed Daubechies filters, since they yield orthonormal expansions that can be implemented exactly by discrete-time filter banks, which ensures stable and perfect reconstruction and moreover that all the information in the processed EEG and EMG signals is equally represented in the SWT domain. Specifically, the Daubechies D4 wavelets were chosen for their balanced time–frequency resolution. The embedding dimension was set to d=8 for all sub-band components of EEG and EMG signals, and the minimum embedding delays $\tau_{j}^{\left(0\right)}$ equal to 8, 4, 2 and 1 sampling points in the delta/theta, alpha, beta and low gamma bands, respectively, were applied. The timescale factor s=4 was selected which means that the temporal context over which interactions were captured ranged from 28–224 ms, depending on the frequency band. The value of s=4 was chosen since this generally gave the strongest MWTE values in our previous study.^37^ The interaction delay was set to 25 ms, in agreement with the underlying neurophysiology^49^ and verified via a delay estimation algorithm based on maximizing TE.^50^ Consistent with the prior study,^37^ we also explored the timescale factor s=1. However, the results corresponding to this factor revealed no significant differences between the groups after adjusting for multiple comparisons. As such, these findings will not be further discussed in the context of this study.

### Estimation

Estimating the TE from a finite number of samples of the time series of interest is a complex task.^51^ Let $x^{-}≜x_{\;j_{x},t-1}^{\tau_{\;j_{x}},d_{x}}$, $y^{-}≜y_{\;j_{y},t+u-1}^{\tau_{\;j_{y}},d_{y}}$, and $y≜y_{\;j_{y},t+u}$, the MWTE (6) can be rewritten as sum of four differential entropies as


(7) $$\begin{array}{rl} \mathrm{MWT}E_{x_{\;j_{x}}\to y_{\;j_{y}}}= & \;I(x_{\;j_{x},t-1}^{\tau_{\;j_{x}},d_{x}};y_{\;j_{y},t+u}|y_{\;j_{y},t+u-1}^{\tau_{\;j_{y}},d_{y}})=H(y,y^{-})\\ & -H(y^{-})-H(y,y^{-},x^{-})+H(y^{-},x^{-}), \end{array}$$

which can then be estimated by combining estimates of the individual differential entropy terms. Following the study in Guo *et al*.,^37^ we use the Kraskov, Stogbauer, Grassberger (KSG) technique^52^ for TE estimation, which builds on the Kozachenko and Leonenko (KL) estimator^53^ of log-probabilities via nearest-neighbour counting. In order to track temporal evolution of MWTE, the whole trial (5 s) was divided into 18 overlapping segments with time shift of 250 ms (256 samples), each lasting 500 ms (512 samples). Then, MWTE was estimated on each segment separately.

As a secondary analysis, following identification of a specific CFC combination of interest, the modulation of the coupling in relation to the stimulus was assessed by comparing the MWTE in the early post-stimulus period with that during the baseline period. The time windows selected for comparison were comparable to those used in our original study.^31^ The difference in MWTE between defined time windows was assessed for each group (dystonia and controls) using the one-sample *t*-test (two-sided). The magnitude of the change in MWTE was also compared directly between the dystonia and control groups using the independent sample *t*-test (two-sided).

### Statistical testing

For each individual, we established the statistical significance of the estimated MWTE against the null hypothesis of zero MWTE. To do this, the MWTE method was applied to independent Gaussian white noise sequences to identify significant values. Specifically, for each individual subject, a total of 1000 pairs of Gaussian independent signals were produced with the variances equal to the variances of investigated neurophysiological signals. The results obtained using these pairs of signals were used to form empirical distributions of MWTE values that correspond to such independent processes.^37^ We then use the 95th percentiles of the respective empirical distributions as the thresholds, which account for ∼95% confidence level. Values larger than these identified thresholds are regarded as significant. To test the hypothesis that patterns of CFC would differ between individuals with dystonia and controls, the proportion of individuals in each group with significant MWTE for a given frequency combination and direction was compared using Fisher's exact test.

Additionally, the difference between the mean level of MWTE in patients and the mean level of MWTE in controls for a given frequency combination and direction (i.e. the absolute levels of MWTE, independent of the significance threshold) was assessed using an independent sample *t*-test. To account for multiple comparisons, the Storey's false discovery rate (FDR) method^54^ was used. Cohen's *d* served as the effect size measure to complement the results from the two-sample *t*-test.

Finally, to investigate the potential confounding effects of the level of EMG (% MVC) and contraction variability of the amplitude of rectified EMG (CV) on the MWTE values, linear regression analyses were performed. These analyses involved the simultaneous consideration of the potential confounding factors (% MVC and CV) and the group assignment as regressors for MWTE values in relevant cross-frequency combinations.

## Results

### Intra-frequency MWTE

**Figure 1:**
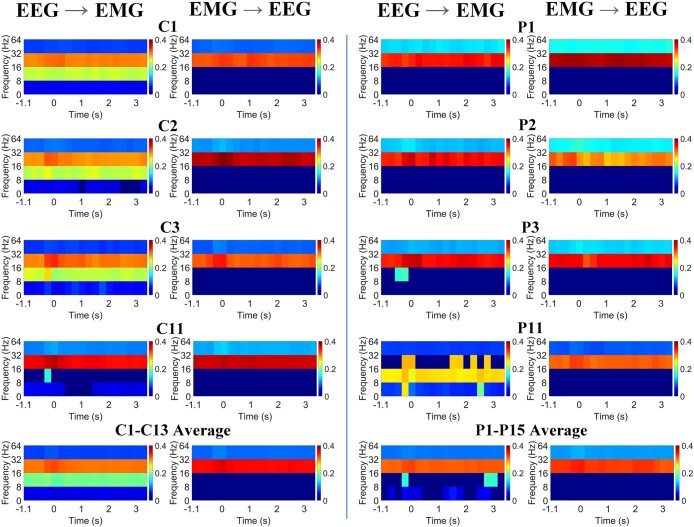
**Intra-frequency multi-scale wavelet transfer entropy (MWTE) comparison between control participants and young people with dystonia.** Rows 1–4 illustrate intra-frequency MWTE in four control participants (C1–C3 and C11) and four children with dystonia (P1–P3 and P11), showing the EEG → EMG and EMG → EEG TE within (32–64) Hz—low gamma, (16–32) Hz—beta, (8–16) Hz—alpha and (0–8) Hz—delta/theta frequency bands over time. The horizontal axis shows time in seconds with the stimulus being time zero, and the vertical axis shows frequency. Gaussian white noise signals were employed to generate empirical distributions of intra-frequency MWTE values, mirroring independent processes. The thresholds for significance were determined by the 95th percentiles of the respective empirical distributions, effectively representing ∼95% confidence intervals. Any values falling below these established thresholds are regarded as non-significant and are consequently assigned a value of zero, visually depicted by the colour dark blue. Significant MWTE is mainly present in the beta range for both directions in all participants, and in the alpha band for the EEG → EMG direction in C1–C3, C11, P3 and P11. Minor fluctuations can be observed over time and in relation to the stimulus (time zero). Row 5 presents the average of intra-frequency MWTE across all participants in each group. This average is computed by considering the absolute intra-frequency MWTE values regardless of significant level, followed by calculating a group threshold calculated as the average of the 95% confidence intervals across all participants in each group. Again, for purposes of illustration, values below this threshold are considered non-significant and set to zero, visually represented by the colour dark blue.

Significant intra-frequency MWTE was detected in all participants, both controls and those with dystonia. In Fig. 1 (illustrative examples from both groups and group averages) and Supplementary Fig. 1 (data from all individuals), it can be observed that significant beta (16–32 Hz) range MWTE was identified in both directions, and was present throughout the epoch, with some minor fluctuations over time and in relation to the stimulus. Significant MWTE was also seen in the 8–16 Hz range for the EEG → EMG direction in most participants, across the epoch (12/13 healthy controls and 10/15 children with dystonia, Supplementary Fig. 1). However, this was not seen in the EMG → EEG direction in either group. When present, the 8–16 Hz MWTE from EEG to EMG was generally less strong and showed more fluctuation than the beta-range MWTE, except in a couple of participants, where it appeared as the dominant frequency (Supplementary Fig. 1). For the intra-frequency MWTE, statistical testing revealed no significant difference between the control and dystonia groups, either on comparing the number of participants showing significant levels of MWTE or comparing the mean level of MWTE between groups (Tables 2 and 3).

**Table 2:** Comparison of the proportion of individuals in each group with significant multi-scale wavelet transfer entropy (MWTE)

EEG (horizontal axis) → EMG (vertical axis)	**Control participants**					EMG (horizontal axis) → EEG (vertical axis)	**Control participants**				
	**32–64**	13/13 (100%)	13/13 (100%)	13/13 (100%)	*13/13 (100%)*		**32–64**	13/13 (100%)	13/13 (100%)	13/13 (100%)	*13/13 (100%)*
	**16–32**	3/13 (23%)	8/13 (62%)	*12/13 (92%)*	13/13 (100%)		**16–32**	**11/13 (85%)**	13/13 (100%)	*13/13 (100%)*	13/13 (100%)
	**8–16**	11/13 (85%)	*10/13 (77%)*	0/13 (0%)	13/13 (100%)		**8–16**	0/13 (0%)	*0/13 (0%)*	0/13 (0%)	13/13 (100%)
	**0–8**	*7/13 (54%)*	12/13 (92%)	2/13 (15%)	13/13 (100%)		**0–8**	*0/13 (0%)*	0/13 (0%)	8/13 (62%)	8/13 (62%)
	**Freq**	**0–8**	**8–16**	**16–32**	**32–64**		**Freq**	**0–8**	**8–16**	**16–32**	**32–64**
	**Young people with dystonia**						**Young people with dystonia**				
	**32–64**	15/15 (100%)	15/15 (100%)	15/15 (100%)	*15/15 (100%)*		**32–64**	15/15 (100%)	15/15 (100%)	15/15 (100%)	*15/15 (100%)*
	**16–32**	2/15 (13%)	11/15 (73%)	*12/15 (80%)*	15/15 (100%)		**16–32**	**2/15 (13%)**	11/15 (73%)	*14/15 (93%)*	15/15 (100%)
	**8–16**	8/15 (53%)	*8/15 (53%)*	1/15 (7%)	15/15 (100%)		**8–16**	1/15 (7%)	*0/15 (0%)*	0/15 (0%)	15/15 (100%)
	**0–8**	*6/15 (40%)*	11/15 (73%)	5/15 (33%)	15/15 (100%)		**0–8**	*0/15 (0%)*	2/15 (13%)	9/15 (60%)	11/15 (73%)
	**Freq**	**0–8**	**8–16**	**16–32**	**32–64**		**Freq**	**0–8**	**8–16**	**16–32**	**32–64**
	**Fisher's exact test (*P-*value)**						**Fisher's exact test (*P*-value)**				
	**32–64**	1.0000	1.0000	1.0000	*1.0000*		**32–64**	1.0000	1.0000	1.0000	*1.0000*
	**16–32**	0.6389	0.6891	*0.6000*	1.0000		**16–32**	**0.0004**	0.1016	*1.0000*	1.0000
	**8–16**	0.1145	*0.2543*	1.0000	1.0000		**8–16**	1.0000	*1.0000*	1.0000	1.0000
	**0–8**	*0.7051*	0.3333	0.3955	1.0000		**0–8**	*1.0000*	0.4841	1.0000	0.6891
	**Freq**	**0–8**	**8–16**	**16–32**	**32–64**		**Freq**	**0–8**	**8–16**	**16–32**	**32–64**
	**FDR-adjusted *P-*value (*q-*value)**						**FDR-adjusted *P*-value (*q*-value)**				
	**32–64**	1.0000	1.0000	1.0000	*1.0000*		**32–64**	0.9546	0.9546	0.9546	*0.9546*
	**16–32**	1.0000	1.0000	*1.0000*	1.0000		**16–32**	**0.0061**	0.7759	*0.9546*	0.9546
	**8–16**	1.0000	*1.0000*	1.0000	1.0000		**8–16**	0.9546	*0.9546*	0.9546	0.9546
	**0–8**	*1.0000*	1.0000	1.0000	1.0000		**0–8**	*0.9546*	0.9546	0.9546	0.9546
	**Freq**	**0–8**	**8–16**	**16–32**	**32–64**		**Freq**	**0–8**	**8–16**	**16–32**	**32–64**

Summary of number of individuals showing significant cross-frequency coupling (CFC), in either direction: EEG → EMG (left table) and EMG → EEG (right table), during the baseline period [−1.1, −0.6] s. For each sub-table, the horizontal frequency axis represents the source, whereas the vertical axis represents the destination. The intra-frequency coupling combinations are shown in italics. A Fisher's exact test was applied to compare the proportions of individuals in each group showing significant coupling for each CFC combination. Storey's FDR method was used to account for multiple comparisons, and the hypothesis testing error measures (*q*-values) were adjusted accordingly. Highlighted in bold: *q* < 0.05.

**Table 3:** Comparison of the mean level of multi-scale wavelet transfer entropy (MWTE) between groups

EEG (horizontal axis) → EMG (vertical axis)	**Two-sample *t*-test (*P*-value)**					EMG (horizontal axis) → EEG (vertical axis)	**Two-sample *t*-test (*P*-value)**				
	**32–64**	0.1795	0.0315	0.0234	*0.0339*		**32–64**	0.3608	0.9663	0.3049	*0.0405*
	**16–32**	0.3152	0.7525	*0.6049*	0.4149		**16–32**	**0.0024**	**0.0031**	*0.1484*	0.0857
	**8–16**	0.0135	*0.0599*	0.2735	0.0639		**8–16**	0.2175	*0.2792*	0.0501	0.1151
	**0–8**	*0.6125*	0.3941	0.2628	0.0890		**0–8**	*0.1045*	0.2422	0.4671	0.7035
	**Freq**	**0–8**	**8–16**	**16–32**	**32–64**		**Freq**	**0–8**	**8–16**	**16–32**	**32–64**
	**FDR-adjusted *P-*value (*q-*value)**						**FDR-adjusted *P*-value (*q*-value)**				
	**32–64**	0.2834	0.1429	0.1429	*0.1429*		**32–64**	0.3799	0.7631	0.3464	*0.1463*
	**16–32**	0.3464	0.6134	*0.5337*	0.4032		**16–32**	**0.0397**	**0.0397**	*0.2500*	0.1873
	**8–16**	0.1138	*0.1615*	0.3359	0.1615		**8–16**	0.3234	*0.3359*	0.1582	0.2078
	**0–8**	*0.5337*	0.3984	0.3359	0.1873		**0–8**	*0.2032*	0.3359	0.4372	0.5926
	**Freq**	**0–8**	**8–16**	**16–32**	**32–64**		**Freq**	**0–8**	**8–16**	**16–32**	**32–64**
	**Two-sample effect size (Cohen's *d*)**						**Two-sample effect size (Cohen's *d*)**				
	**32–64**	−0.5075	−0.8362	−0.8862	−*0.8236*		**32–64**	0.3422	0.0157	−0.3850	−*0.7931*
	**16–32**	0.3767	−0.1172	−*0.1927*	−0.3048		**16–32**	**1.2364**	**1.1976**	*0.5479*	−0.6573
	**8–16**	0.9750	*0.7239*	0.4115	0.7120		**8–16**	0.4649	*0.4066*	0.7559	−0.5997
	**0–8**	*0.1886*	0.3188	−0.4210	0.6500		**0–8**	−*0.6188*	−0.4403	0.2715	−0.1416
	**Freq**	**0–8**	**8–16**	**16–32**	**32–64**		**Freq**	**0–8**	**8–16**	**16–32**	**32–64**

Comparison of the mean level of MWTE in the control group and the mean value of MWTE in the dystonia group, in either direction: EEG → EMG (left table) and EMG → EEG (right table), during the baseline period [−1.1, −0.6] s. For each sub-table, the horizontal frequency axis represents the source, whereas the vertical axis represents the destination. The intra-frequency coupling combinations are shown in italics. A two-sample *t*-test is applied to investigate whether the unknown population means of two groups are statistically equal or not. Storey's FDR method was used to account for multiple comparisons, and the hypothesis testing error measures (*q*-values) were adjusted accordingly. Cohen's *d* was employed as a measure of effect size to accompany reporting of two-sample *t*-test results. Highlighted in bold: *q* < 0.05.

### Cross-frequency MWTE

**Figure 2:**
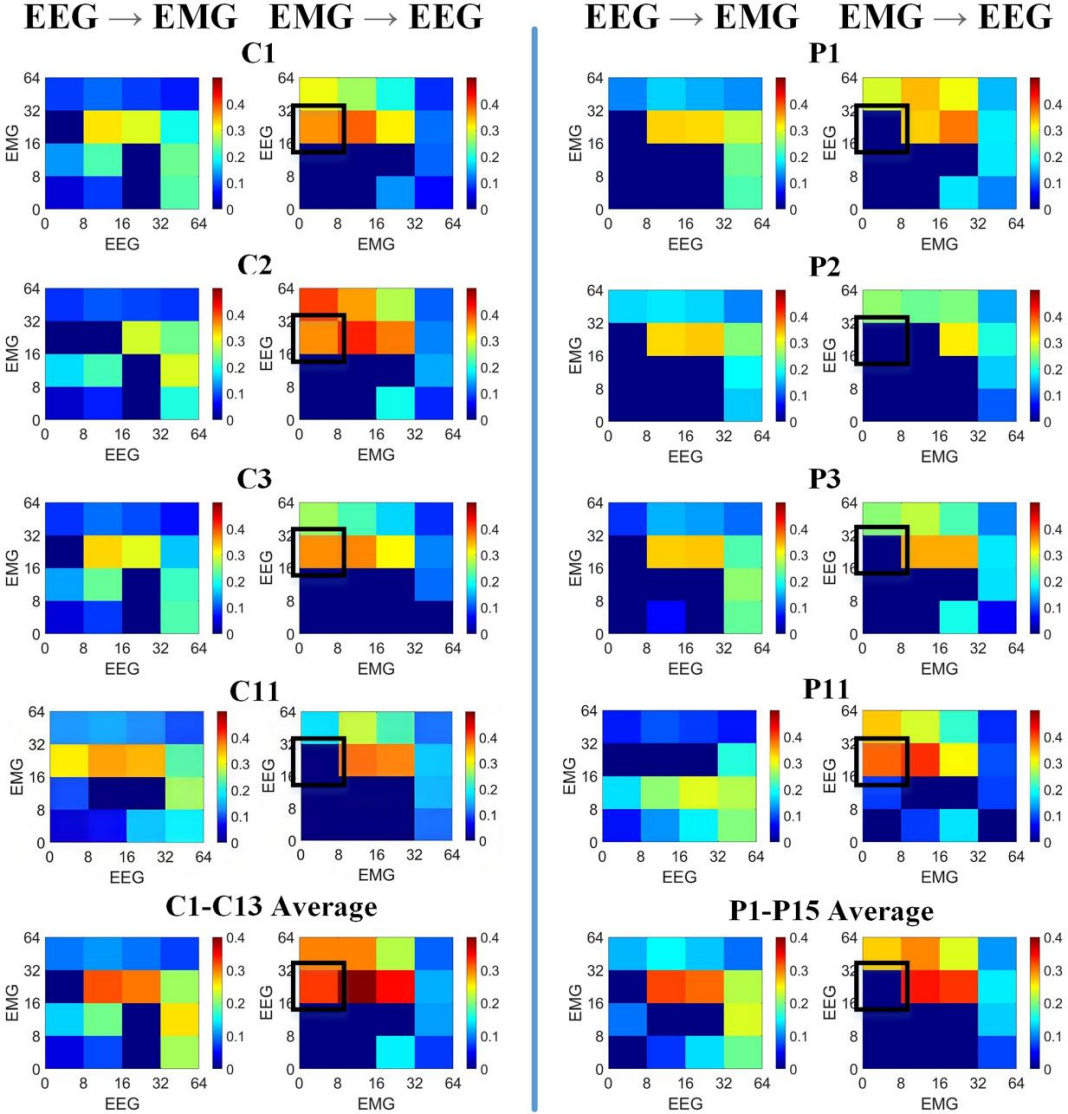
**Cross-frequency multi-scale wavelet transfer entropy (MWTE) comparison between control participants and young people with dystonia.** Rows 1–4 illustrate cross-frequency MWTE in four control participants (C1–C3 and C11) and four children with dystonia (P1–P3 and P11) for both directions, focusing on four functional bands, delta/theta (0–8) Hz, alpha (8–16) Hz, beta (16–32) Hz and low gamma (32–64) Hz, in the baseline period [−1.1, −0.6] s. The horizontal axis represents the source, whereas the vertical axis represents the destination. Gaussian white noise signals were employed to generate empirical distributions of cross-frequency MWTE values, mirroring independent processes. The thresholds for significance were determined by the 95th percentiles of the respective empirical distributions, effectively representing ∼95% confidence intervals. Any values falling below these established thresholds are regarded as non-significant and are consequently assigned a value of zero, visually depicted by the colour dark blue. The cross-frequency couplings generally exhibit similar patterns between the two groups, except that the control group displayed a pronounced interaction from the EMG (0–8 Hz) band to the EEG beta (16–32 Hz) band that was rarely seen in the dystonia group. Row 5 presents the average of cross-frequency MWTE across all participants in each group. This average is computed by considering the absolute cross-frequency MWTE values regardless of significant level, followed by calculating a group threshold calculated as the average of the 95% confidence intervals across all participants in each group. Again, for purposes of illustration, values below this threshold are considered non-significant and set to zero, visually represented by the colour dark blue.

CFC analysis revealed significant MWTE across multiple frequency bands. Figure 2 shows illustrative examples of the CFC-MWTE analysis during the baseline period [−1.1, −0.6] s for four controls and four children with dystonia. The group averages are also included. Plots for the other participants are shown in Supplementary Fig. 2. Table 2 summarizes the percentage of all individuals tested (C1–C13 and P1–P15) showing significant CFC for each frequency combination, in the baseline period [−1.1, −0.6] s, along with results of Fisher's exact tests comparing the proportions of individuals in each group showing significant coupling for each combination. For most CFC combinations, the MWTE patterns are largely similar between the two groups. However, controls tend to show a strong communication from the EMG (0–8 Hz) range to the EEG beta (16–32 Hz) range, seen in 11/13 participants, which was seen only rarely in children with dystonia (2/15). This difference is statistically significant (Fisher's exact test *P* = 0.0004) and remained significant after correcting for multiple comparisons (*q* = 0.0061) (Table 2).

The mean value of MWTE for each CFC combination (independent of the significant threshold) was also compared between groups using the independent sample *t*-test. The corresponding results during the baseline period [−1.1, −0.6] s are shown in Table 3. CFC from EEG to EMG is not statistically different between groups. For the direction from EMG to EEG, patients with dystonia again show significantly less CFC from delta/theta (0–8 Hz) EMG to beta (16–32 Hz) EEG (*q* < 0.05, Cohen's *d* = 1.2364), similar to the analysis above. In addition, the dystonia group show less coupling from alpha (9–16 Hz) EMG to beta (16–32 Hz) EEG (*q* < 0.05, Cohen's *d* = 1.1976).

It is noted that the Fisher exact test only compares the proportions of participants showing significant coupling for a given CFC combination, whereas using the *t*-test to evaluate the absolute level of MWTE is more sensitive, allowing comparison of numerical values between groups even if these are both above chance level. In this case, it demonstrates that the alpha-EMG to beta-EEG CFC combination is reduced in dystonia compared with controls: in some cases, this reduction is below the significance level while in others, coupling is detected above the significance level but with values still relatively lower than those seen in the control group.

Within the dystonia group, no significant correlation was identified between the magnitude of coupling from low-frequency EMG to beta EEG and the participants’ BFMDRS-m scores (Pearson *R* = −0.049, *P* = 0.887) or BFMDRS-d scores (Pearson *R* = −0.176, *P* = 0.604).

### Temporal evolution of MWTE

**Figure 3:**
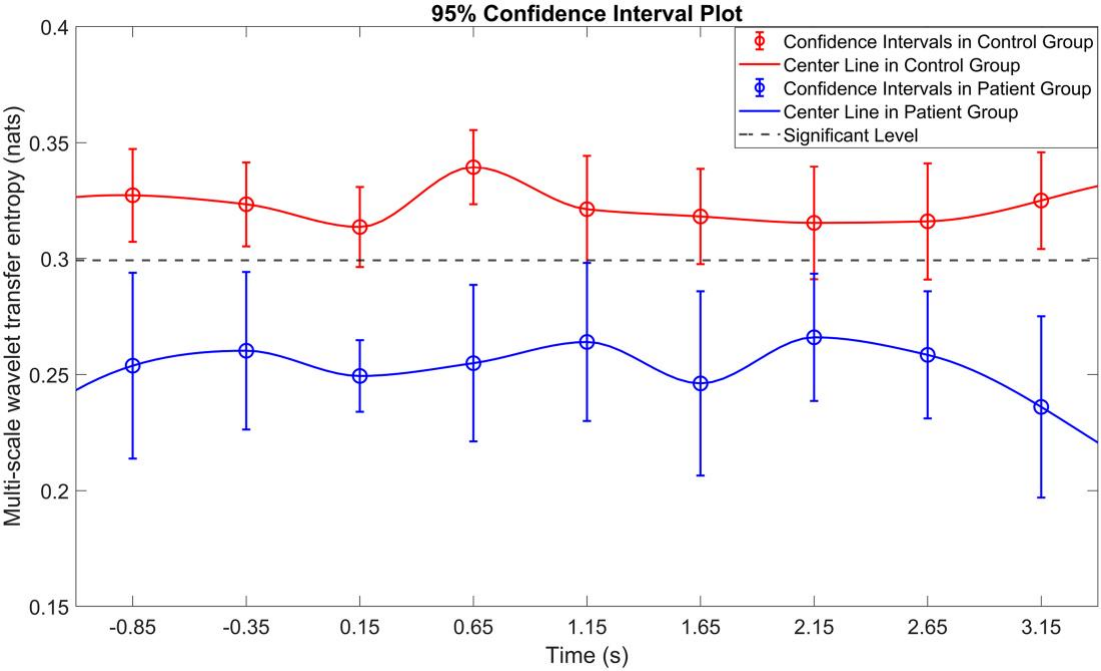
**Temporal evolution of cross-frequency multi-scale wavelet transfer entropy (MWTE).** The mean value of MWTE with 95% confidence intervals in control and dystonia groups over time, focusing on the frequency combination from EMG delta/theta (0–8) Hz to EEG beta (16–32) Hz. The horizontal axis shows time in seconds with the stimulus being time zero, the vertical axis shows the TE value. The mean value of MWTE in each time window and group is drawn with the circle marker, with vertical bars showing the 95% confidence intervals. The centre line is a vector of interpolated values corresponding to the mean values of MWTE in nine non-overlapping time windows. Gaussian white noise signals were employed to generate empirical distributions of cross-frequency MWTE values that correspond to independent processes. The 95th percentiles of the respective empirical distributions were used as thresholds, representing ∼95% confidence intervals, and are shown by the dotted line. It is noted that the mean value of MWTE in the dystonia group falls below the significance/chance level in all time windows. The difference between controls and dystonia is maintained across the time window. A discernible degree of modulation is observed following the mechanical perturbation in controls, characterized by a significant increase in MWTE during time window 4 as compared to time windows 1–2 (one-sample *t*-test, *t* = 3.520, *P* = 0.004). In contrast, this modulation is markedly diminished in the dystonia group (*t* = −0.530, *P* = 0.604).

The cross-frequency coupling MWTE was calculated for each of the nine non-overlapping time windows across the epoch. Figure 3 plots the MWTE from EMG delta/theta (0–8) Hz to EEG beta (16–32) Hz over time, showing the mean and 95% confidence intervals for each group. The control groups show significant MWTE in this CFC combination across the entire epoch whereas the mean value of MWTE in the dystonia group remains below the chance level.

Thus, the difference between controls and dystonia is maintained across the epoch. Individual plots for the early post-stimulus period are shown for each participant in the supplementary information, together with the corresponding statistical comparisons (Supplementary Fig. 3 and Supplementary Tables 1 and 2).

Figure 3 also indicates a modulation of the coupling between EMG delta/theta and EEG beta following the stimulus. The difference in the level of MWTE from the baseline period (time windows 1–2) to the early post-stimulus period (time window 4) was significant for the control group (one-sample *t*-test, *t* = 3.520, *P* = 0.004), but not for the dystonia group (*t* = −0.530, *P* = 0.604). The difference between groups in the magnitude of the change in MWTE for this frequency combination was significant (independent sample *t*-test, *t* = 2.842, *P* = 0.009).

### Consideration of potential confounding factors

The task involved isometric contraction, and the stability in the level of muscle contraction [assessed as coefficient of variation (CV) of the amplitude of the EMG] was used as a measure of task performance.^31^ As noted in the original study,^31^ at group level, individuals with dystonia tended to use a higher % of maximum voluntary contraction (% MVC) to perform the task compared with controls (mean % MVC for FDI: controls 1.531, dystonia 3.985, *t* = −4.267, *P* = 0.001). However, on linear regression analysis, the % MVC does not emerge as a significant confounding factor on individual levels of MWTE from the EMG (0–8 Hz) range to the EEG (16–32 Hz) range, with an insignificant association between % MVC and MWTE (*P* > 0.05) throughout the entire epoch. Similarly, the CV does not operate as a confounding factor on individual levels of MWTE for the same frequency combination, with linear regression consistently revealing an insignificant association between CV and MWTE (*P* > 0.05). In contrast, a significant association was observed between group assignment (controls versus dystonia) and MWTE (*P* < 0.05) in 7 out of 9 time windows, as detailed in Supplementary Table 3.

## Discussion

TE is an established method for identifying both linear and non-linear coupling between two processes,^37,43,44,55^ and the particular method of MWTE allows the assessment of interactions on multiple time scales and across frequency bands.^37^ To our knowledge, this is the first study to apply MWTE to investigate cortex–muscle interactions in dystonia. The key findings are:

1. Significant bidirectional interactions are demonstrated between cortex and muscle in individuals with dystonia during a motor task, comparable to healthy controls (Fig. 1).
2. Individuals with dystonia show a specific abnormality of cross-frequency coupling with significantly weaker communication from low-frequency (0–8 Hz) EMG to beta-range (16–32 Hz) EEG compared with controls (Fig. 2 and Tables 2 and 3). In addition, the stimulus-related modulation of this specific cross-frequency coupling interaction seen in controls is impaired in dystonia.
3. Cross-frequency coupling from alpha range (9–16 Hz) EMG to beta-range (16–32 Hz) EEG was also diminished in dystonia compared with controls, although this was a less striking difference.

These novel findings enhance scientific knowledge of dystonia pathophysiology and provide a more detailed understanding of how sensorimotor feedback is disrupted in individuals with dystonia.

###  

#### MWTE demonstrates bidirectional cortex–muscle interactions in dystonia

While it is evident from clinical observation that cortex–muscle interactions must be present in individuals with dystonia, spectral analysis methods used hitherto have often found it difficult to demonstrate these,^26,31^ particularly for beta-range CMC. A similar conundrum is noted even in healthy individuals, a proportion of whom do not show significant levels of beta-range CMC during a steady grasp task despite having normal motor control.^45,49,56^ We demonstrated previously that healthy adults who do not manifest significant CMC during a baseline modest contraction do show significant CMC following a sensory stimulus relevant to the task.^45^ This was also the case in healthy children/teenagers.^31^ However, in young people with dystonia performing the same paradigm, levels of CMC remained low throughout the task and the stimulus-related increase of beta-CMC observed in healthy individuals was not seen, particularly in those with genetic or idiopathic dystonia aetiologies.^31^ We interpreted this finding as evidence of impaired sensorimotor integration in dystonia. However, the generally low levels of cortex–muscle interactions detected in dystonia with standard coherence methods^26,31^ have remained a barrier to a more thorough understanding of the underlying sensorimotor pathophysiology. Therefore, the current demonstration of significant bidirectional communication between cortex and muscle in individuals with dystonia using the MWTE method is an important advance and illustrates the value of using multiple techniques to explore the underlying neural pathways.

Some elements of neural transmission take place, at least in part, in a linear manner. This means that linear methods such as standard coherence and Granger causality/directed coherence can still reveal some important aspects of neural behaviour. For example, in the monosynaptic transmission from cortex to motoneurons via the corticospinal tract, the spread of a common input to the motoneuron pool effectively smooths out the non-linearity of individual neurons,^38,57^ resulting in an effectively linear communication between cortex and muscle, as demonstrated by the presence of corticomuscular coherence. Directed coherence measures such as Granger causality add information on the direction of cortex–muscle interactions,^33^ but both CMC and Granger causality are limited in detecting only linear interactions between brain and muscle.^37^ If used alone, the delineation of neural systems using these methods is incomplete.^38^ MWTE confers the additional advantage of allowing detection of both linear and non-linear coupling,^37^ with non-linear interactions detectable both across and within frequency bands. In this context, clear cortex–muscle interactions are revealed in the dystonia group. One could postulate that this indicates a relatively higher proportion of non-linear cortex–muscle interactions in the dystonia group, perhaps involving polysynaptic pathways, whereas the linear aspect of their cortex–muscle interactions may be impaired. Central motor conduction times are normal in the majority of young people with dystonia indicating integrity of the corticospinal tract,^58,59^ but the linear communication from cortex to muscle in the beta frequency range, which appears to relate to maintaining a steady force,^60,61^ is disrupted in this population.^26,31^

Interestingly, although linear interactions have rarely been demonstrated between cortex and muscle in dystonia, there is clear evidence of coherence between ‘sub-cortical’ sources of neuronal activity and dystonic EMG. For example, using the directed transfer function, Sharott *et al.*^23^ demonstrated bidirectional coupling between low-frequency (<10 Hz) local field potentials in the globus pallidus internus (GPi) and dystonic sternocleidomastoid EMG in patients with cervical dystonia, with the stronger direction of interaction being from GPi to muscle. Neumann *et al.*^62^ found significant coherence in the 4–7 Hz band between the subthalamic nucleus and dystonic EMG. Both these findings would suggest predominantly linear (although most likely indirect) forms of coupling.^23^ Due to the invasive nature of these recordings, it is not possible to determine how these findings compare with interactions between sub-cortical structures and EMG in healthy individuals. However, the observation that coherence between low-frequency GPi oscillatory activity and dystonic EMG is suppressed by pallidal deep brain stimulation and relates to clinical phenotype^24^ suggests that the phenomenon is clinically relevant.

In this context, it is also important to note the abnormally enhanced low-frequency ‘intermuscular’ coherence (IMC) observed in individuals with dystonia of genetic or idiopathic origin^26-28,30,31^ or of acquired aetiology.^31^ The observations that this phenomenon is present across multiple aetiologies of dystonia and that it correlates with dystonia severity^30,31^ suggest that it represents a functional mechanistic process. While intermuscular coherence has often been used to infer patterns of cortical drive to muscle, there is evidence that IMC cannot be taken as a pure surrogate marker of CMC.^31^ While there is likely to be some overlap, IMC may reflect, at least in part, sub-cortical processes. For example, in this context, the low-frequency IMC seen in dystonia might reflect an increased low-frequency linear drive from GPi to muscle as observed by Sharott *et al.*^23^ The route of coupling from pallidum to dystonic muscle could not be determined in that study, but the authors point out that it would likely involve either input to the thalamus or motor cortex and/or input to brainstem relays to motor structures.^23^ The possibility also remains that the coherence between GPi and muscle reflects another common source driving both GPi and muscle, but reaching the GPi first, hence the apparent directional drive from GPi to muscle.

#### Cross-frequency coupling reveals aspects of cortex–muscle communication not detected by linear coherence methods

Cross-frequency coupling within the nervous system has gained considerable interest in recent years and is thought to play a crucial role in communication between distant areas and the integration of information across multiple spatiotemporal scales.^63,64^ For example, a dynamic causal modelling study based on MEG data recorded during a grasping task provided evidence that intra-cortical cross-frequency coupling in the human motor system occurred predominantly between different nodes of the distributed neural network, whereas coupling intrinsic to a given area was predominantly iso-frequency and linear.^65^ Another putative function is stimulus parsing, with evidence that coupling between theta and gamma oscillations within the cortex is involved in the parsing of speech.^63^ Cross-frequency interaction between beta and theta activities in the subthalamic nucleus has also been demonstrated in patients with Parkinson's disease.^66^

While cross-frequency coupling is often studied in the context of intra-cortical or cortico-sub-cortical communication,^63-65,67-70^ cross-frequency coupling between cortex and muscle has been less extensively explored.^37,71,72^ There is some recent work applying transfer entropy-based techniques to understand corticomuscular coupling in stroke^42,73^ but to our knowledge, this is the first report of CFC between cortex and muscle in individuals with dystonia. In both our current and previous studies using MWTE,^37^ we found that, in healthy individuals, although CFC was present in both directions, coupling from EEG to EMG was more often seen ‘within’ a given frequency band (e.g. alpha–alpha or beta–beta) whereas coupling from EMG to EEG was more often seen ‘across’ frequency bands. In particular, lower EMG frequencies (theta and alpha) tend to feed back to higher EEG frequencies such as beta.^37^ This is concordant with other reports in healthy individuals indicating that CFC is a more prominent mode of communication in the sensory/afferent arm of the pathway than in the motor/efferent arm,^71,74^ and is in keeping with the underlying dynamics of sensory and motor pathways. As noted by Yang *et al.*,^71^ the afferent pathway from periphery to cortex is less direct and involves a higher number of synapses than the corticospinal tracts: after the initial generation by the muscle of changes in force, length and position, the pathway involves encoding of this sensory/proprioceptive information by receptors such as muscle spindles or Golgi tendon organs, which is then transmitted via their respective afferent nerve fibres, a synaptic relay in the dorsal column nuclei and another in the thalamus, before 3rd order neurons finally pass via the thalamocortical radiation to the somatosensory cortex. The input–output function of synapses is not linear^75^ and the neuronal processing of synaptic inputs can result in modulation of inter-spike intervals, with the consequence that each synapse increases the opportunity for non-linear transmission and cross-frequency coupling.^39^ Hence communication in afferent, sensory pathways is likely to comprise a greater proportion of cross-frequency interaction than in efferent, motor pathways.^74^

Notwithstanding this, transmission in multi-synaptic descending motor pathways such as the cortico-bulbospinal tracts also favours a higher proportion of cross-frequency rather than iso-frequency coupling.^39^ Thus, in order to fully evaluate communication within sensory and motor pathways in health and disease, it is essential to incorporate methods capable of detecting both non-linear and linear interactions, and to investigate both cross-frequency and iso-frequency coupling.^74^ As noted above, the observation in the current study of clear bidirectional cortex–muscle communication demonstrated using MWTE, but not with traditional coherence, could be interpreted as indicating a relatively higher proportion of non-linear (versus linear) cortex–muscle interactions in dystonia. This imbalance could in turn reflect a shift in favour of communication via multi-synaptic cortico-bulbospinal pathways at the expense of more direct linear transmission via the corticospinal pathway.

#### MWTE cross-frequency coupling reveals a specific abnormality of sensorimotor feedback in dystonia

In the current study, both intra-frequency and cross-frequency coupling were detected in individuals with dystonia, with generally similar patterns between the dystonia and control groups. However, the strong communication from EMG delta/theta to EEG beta seen in controls was seen very rarely in children with dystonia, regardless of aetiology. This specific finding was highly statistically significant and has a large effect size (Table 3) as well as being persistent across all time windows (Fig. 3), and is consistent with reduced feedback from muscle to cortex in dystonia. There is already clear evidence of abnormal sensory feedback and processing in dystonia,^12-15,31,76,77^ particularly in relation to proprioceptive information,^15,31,78^ but the current finding is novel in demonstrating a distinct abnormality of cross-frequency communication from muscle to cortex, relating to specific frequency bands of neuronal oscillatory activity. There was also evidence of reduced coupling from 8–16 Hz EMG to 16–32 Hz EEG in dystonia, when comparing absolute levels of MWTE between groups (Table 3).

Rhythmic oscillatory activities within cortex and sub-cortical structures are considered to play key functional roles.^79^ For example, cortical beta oscillations are associated with static motor control^79^ and play a role in signal propagation of corticospinal interactions.^80^ Modulation of mu activity (8–13 Hz) over sensorimotor cortex in response to passive or active movement is considered to reflect processing of movement-related afferent information,^81-83^ while cortical theta oscillations appear to relate to cognitive processes^79,80^ or large-scale co-ordination of information processing across brain regions.^64,84^ These proposed functional roles have been reinforced by the observation of key pathophysiological patterns of neuronal oscillations in some neurological disorders: the observation that abnormally enhanced beta oscillations within the basal ganglia relate to bradykinesia/akinesia in Parkinson's disease and are suppressed by therapeutic administration of dopaminergic medication^85^ or deep brain stimulation of the subthalamic nucleus^86^ emphasizes the importance of beta oscillations in motor control.

In dystonia, there is strong evidence for a pathophysiological role of exaggerated theta and alpha range oscillations across the basal ganglia-cortico-cerebellar network:^22^ these activities are coherent across hemispheres^25^ and are reduced by therapeutic pallidal deep brain stimulation.^24,25^ We recently demonstrated that excessive synchronization of theta band oscillatory activity across multiple brain regions in dystonia has a dynamic quality, being triggered in response to a proprioceptive stimulus,^87^ a finding relevant to the dynamic nature of clinical dystonia, which is often triggered by stimuli, movement or even the intention to move. However, that study (as with many others) focused on iso-frequency communication across brain regions, rather than between brain and muscle.

The novel finding in the current study that the specific feedback from theta-range (and to some extent also alpha-range) EMG to beta-range EEG is lacking in dystonia is pertinent when considered in the context of the excessive low-frequency (4–12 Hz) descending drive to muscles seen characteristically in this population.^26-31^ As discussed above, the origin of this drive is not fully established and may in part be sub-cortical, but the observation that individuals with dystonia show impaired feedback of this low-frequency information from muscle to brain compared with controls is notable. We postulate that an impaired feedback of EMG activity conveyed by theta oscillations to cortical processes represented by beta oscillations could lead in turn to an un-tempered low-frequency descending drive to muscle, perhaps mediated via one of the sub-cortical pathways outlined above. Importantly, both these phenomena have been demonstrated in both isolated genetic or idiopathic dystonias, and in individuals with acquired dystonia.^31^ It is also remarkable that this specific cross-frequency combination of low-frequency EMG to beta EEG was modulated by the proprioceptive stimulus in controls but failed to do so in dystonia (Fig. 3). This is consistent with evidence from other studies of impaired sensory feedback using spectral measures in dystonia, including impaired event-related modulation of sensorimotor cortex mu activity in relation to a passive wrist stretch^15^ and impaired modulation of beta-CMC.^31^ It is not yet clear how these observations relate to the excessive event-related cortical theta synchronization seen across multiple brain regions in response to a proprioceptive stimulus,^87^ as mentioned above, but one hypothesis could be that the excessive induced theta synchronization across multiple brain regions somehow blocks the coupling between theta EMG and beta EEG, which in turn impairs the integration of this information into the beta descending drive.^31^ Observations that cortical hypersynchrony during anaesthesia correlates with and precedes loss of late stimulus-related sensory responses and a breakdown of sensory processing^88^ would support this notion.

It is of course not possible to distinguish whether the failure of feedback from theta EMG to beta EEG fuels the exaggerated low-frequency descending drive to EMG, or whether the reduction in feedback of theta EMG to beta EEG reflects a form of compensation for the excessive low-frequency EMG drive. If the latter explanation was true, one might expect a reduction also in feedback of theta or alpha EMG to gamma EEG in dystonia, whereas these interactions appear preserved. We therefore suggest that the former is the more likely explanation, although this cannot be determined from the current data. The broader role of non-linear interactions in the context of dystonia, in which atypical physiology is likely to reflect a combination of underlying aetiological mechanisms and adaptive secondary changes resulting from long-standing dystonic symptoms, is a complex area for future research to address.

#### Possible study limitations and future work

Patient numbers were relatively small, and a sample size calculation was not performed specifically with regards to the MWTE method, since the data required for such a calculation were not available. The sample size was sufficient to demonstrate significant differences between dystonia and controls in patterns of intermuscular and corticomuscular coherence in the original study,^31^ and between patterns of CFC in the current study. Nevertheless, additional more subtle differences between the control and dystonia groups may have gone undetected, and future replication of the findings in larger studies is needed. The findings from the current study will inform sample size calculations to enable such studies to be powered appropriately. Heterogeneity of aetiology is acknowledged, but this can also be considered as a strength since studying patients with different sub-types of dystonia using the same methodology is important for determining common pathophysiological features. We identified similar findings between patients with the genetic, idiopathic or acquired aetiologies listed in Table 1, but it is not possible to conclude that the results would be transferable to all dystonia populations or to participants aged under 12 years. The magnitude of coupling from low-frequency EMG to beta EEG did not correlate significantly with clinical severity (BFMDRS-m). This could reflect the small sample size and/or the fact that all recruited participants were relatively mildly affected, since more severely affected children were unable to perform the task. Another consideration is that the dominant hand was used for performing the motor task and recording the EMG. This included some individuals with an asymmetric dystonia who were unable to perform the task with their more severely affected hand. It is therefore possible that abnormalities in these individuals were under-estimated. A single bipolar EEG derivation was used in this study, which limits our ability to investigate potential cortical re-organization in relation to cortex–muscle interactions. Future studies could include broader coverage of the cortex. It is noted that individuals with dystonia used a slightly higher % MVC to perform the task than controls. However, a regression analysis demonstrated that this was not a significant confounding factor. Finally, there are a number of ways in which the MWTE technique could be developed for future studies, including optimization of the time–frequency resolution, e.g. optimal wavelet transforms.

## Conclusion

Application of a broader range of analytical techniques to interrogate brain–muscle interactions, including assessment of both linear and non-linear and iso- and cross-frequency coupling, is needed to reveal deeper mechanistic insights into both typical sensorimotor development and into the underlying pathophysiology of movement disorders. He *et al.*^89^ demonstrated abnormal non-linear dynamics in the thalamocortical loop in patients with essential tremor, while our own novel observations now reveal a specific impairment of cross-frequency feedback from muscle to brain in dystonia. There is clear potential for greater exploration in this field. The findings here not only provide new and more detailed understanding of impaired sensorimotor feedback in dystonia but also pave the way for more extensive studies of non-linear dynamics in this population, which in turn may inform future protocols for therapeutic neuromodulation.

## Supplementary material

Supplementary material is available at *Brain Communications* online.

## Supplementary Material

fcae061_Supplementary_Data

## Data Availability

Data are available from the corresponding author upon reasonable request.
