# Supplementary material for: Cross-frequency cortex–muscle interactions are abnormal in young people with dystonia
Source: Brain Commun. 2024 Feb 26;6(2):fcae061. doi: 10.1093/braincomms/fcae061 (PMC10939448; doi:10.1093/braincomms/fcae061)
Supplement: fcae061_Supplementary_Data [file fcae061_supplementary_data.pdf]

Cross-frequency cortex-muscle interactions are abnormal in young people with dystonia

Supplementary Information

Supplementary Figure 1 Intra-frequency multi-scale wavelet transfer entropy (MWTE) comparison between control participants and young people with dystonia. Intra-frequency MWTE in 13 control participants (C1-C13) and 15 children with dystonia (P1-P15), showing the EEG → EMG and EMG → EEG TE within (32-64) Hz - low gamma, (16-32) Hz - beta, (8-16) Hz - alpha, and (0-8) Hz - delta\theta frequency bands over time. The horizontal axis shows time in seconds with the stimulus being time zero, the vertical axis shows frequency. Gaussian white noise signals were employed to generate empirical distributions of intra-frequency MWTE values, mirroring independent processes. The thresholds for significance were determined by the 95th percentiles of the respective empirical distributions, effectively representing approximately 95% confidence intervals. Any values falling below these established thresholds are regarded as non-significant and are consequently assigned a value of zero, visually depicted by the colour dark blue.

Intra-frequency MWTE

EEG → EMG **EMG → EEG**

C1

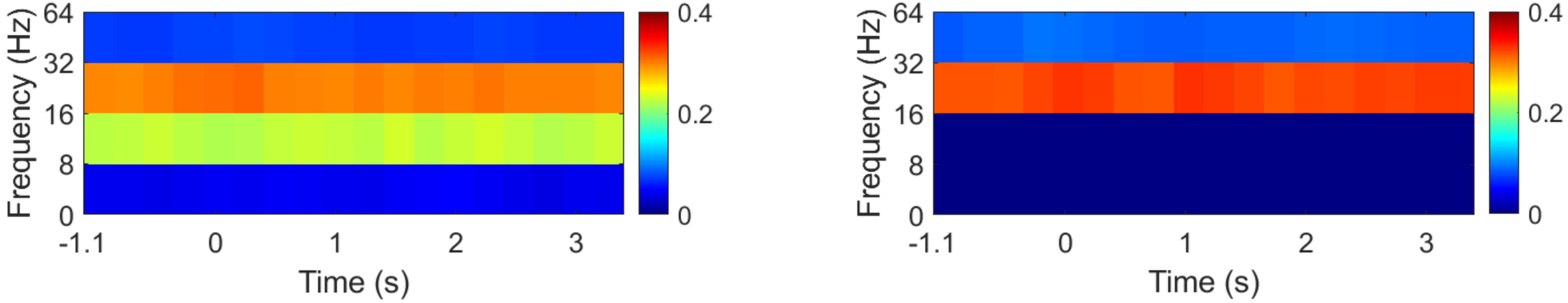

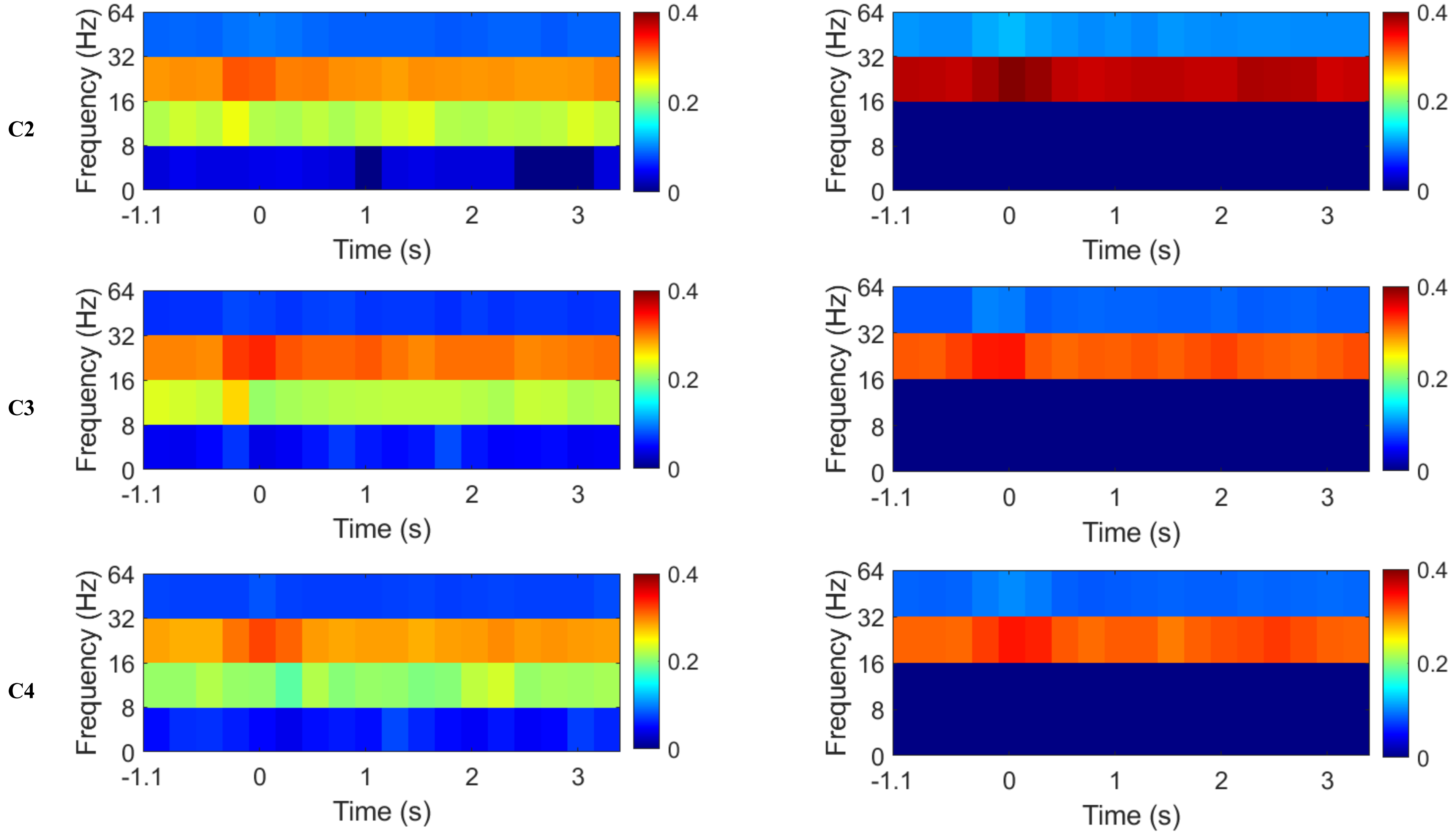
C2
C3
C4
Frequency (Hz)
64
32
16
8
0
Time (s)
-1.1
0
1
2
3
0.4
0.2
0

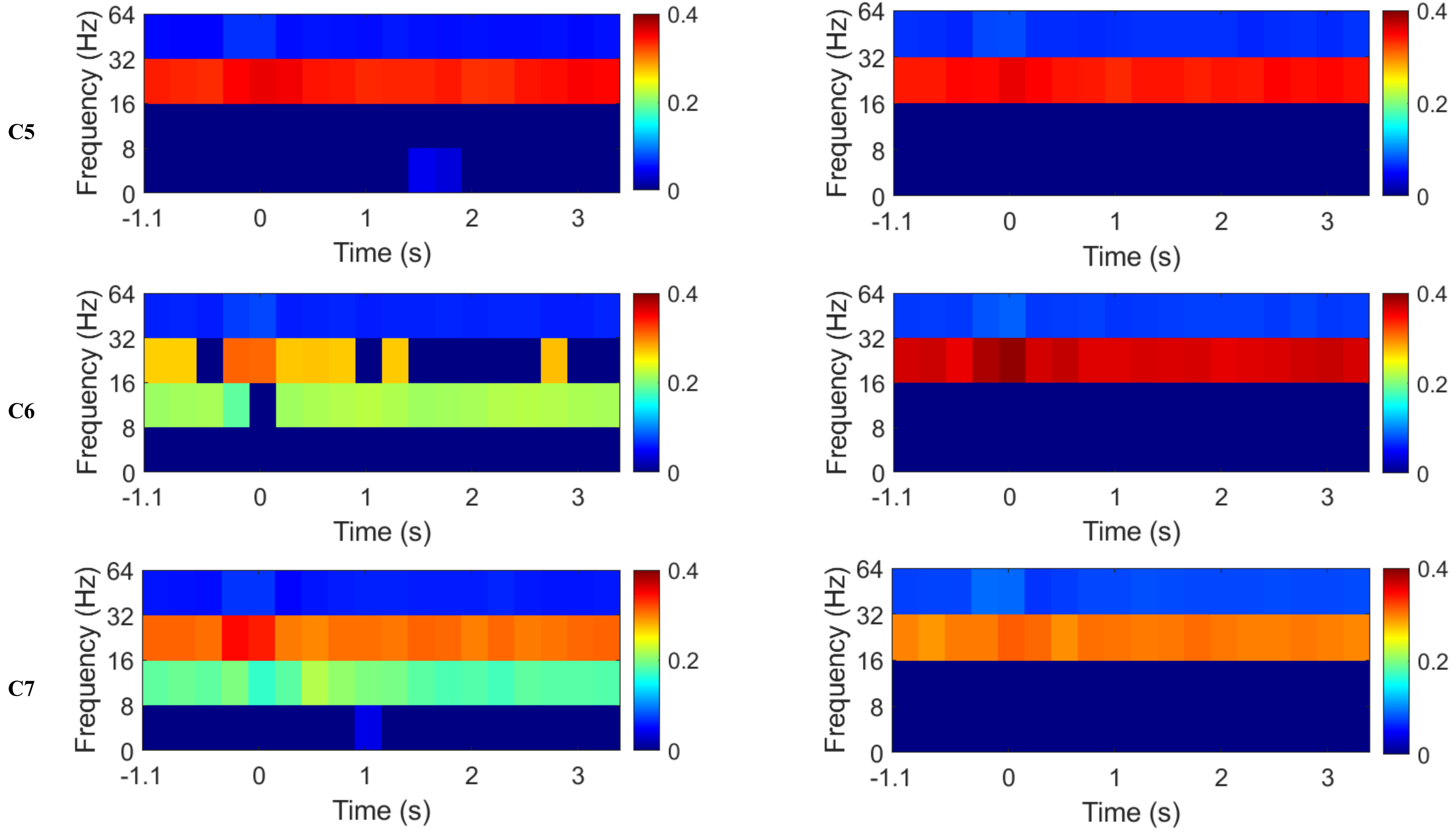
C5
C6
C7
Frequency (Hz)
64
32
16
8
0
Time (s)
-1.1
0
1
2
3
0.4
0.2
0

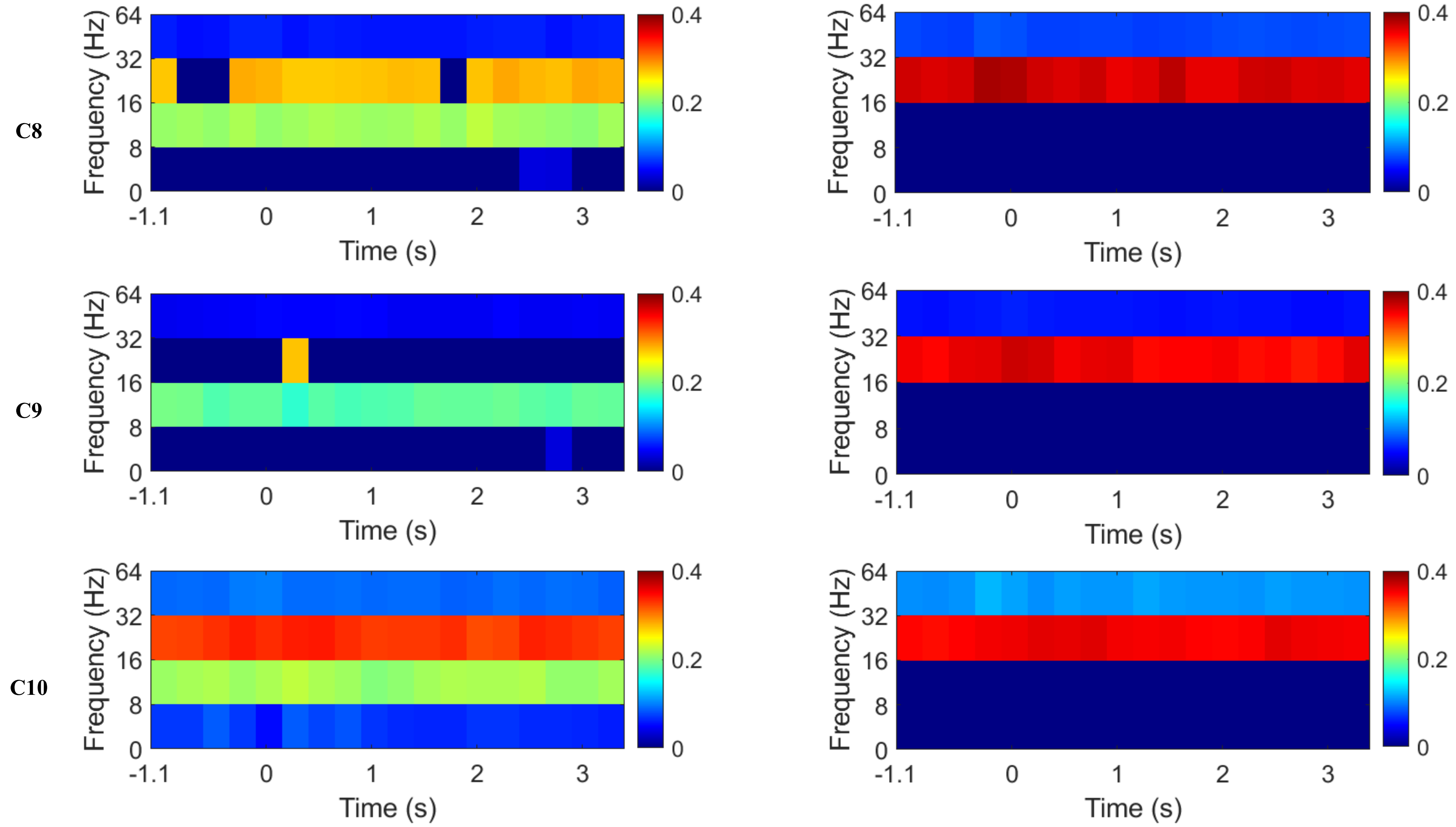
C8
C9
C10
Frequency (Hz)
Time (s)
64
32
16
8
0
-1.1
0
1
2
3
0.4
0.2
0

C11

C12

C13

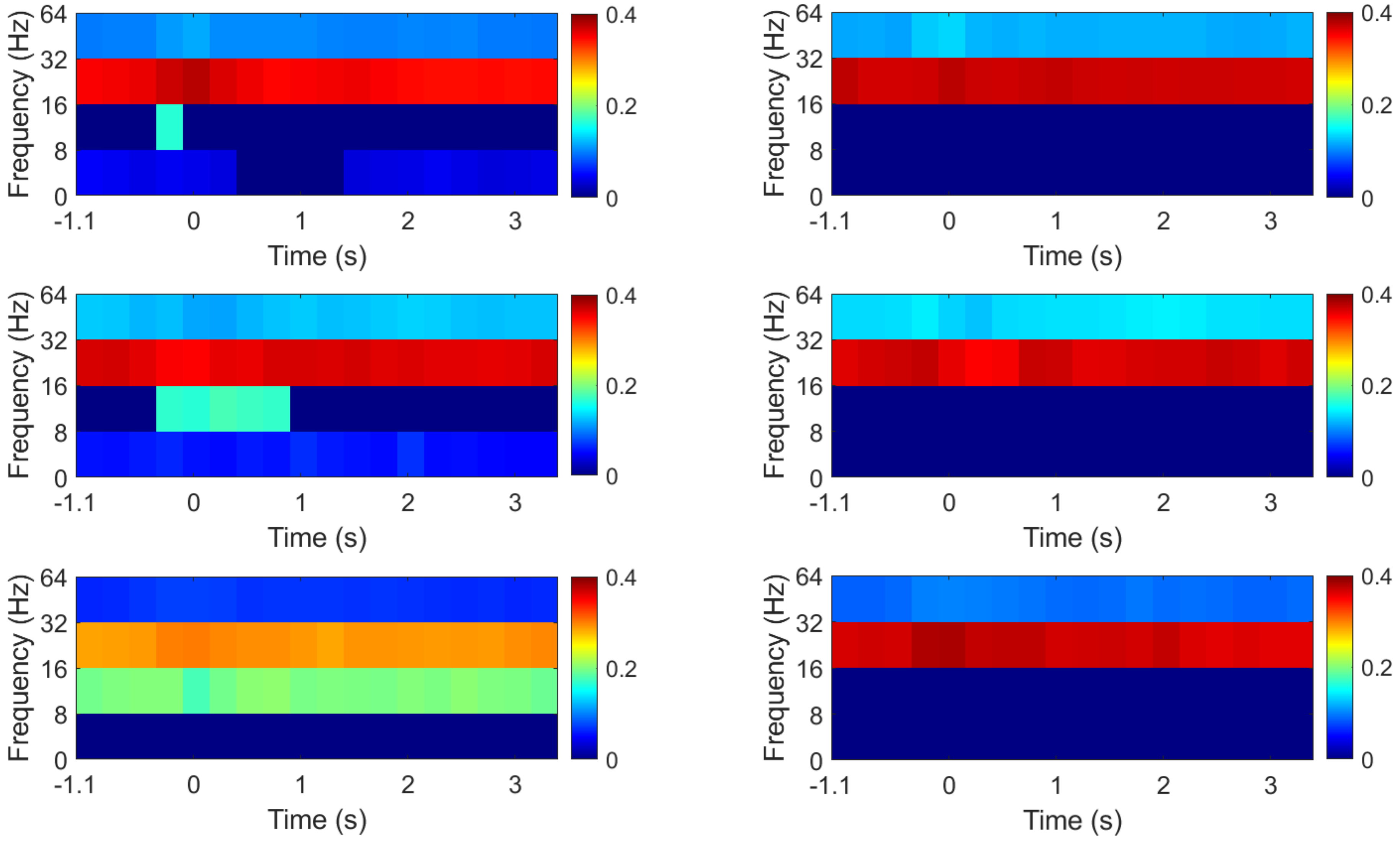

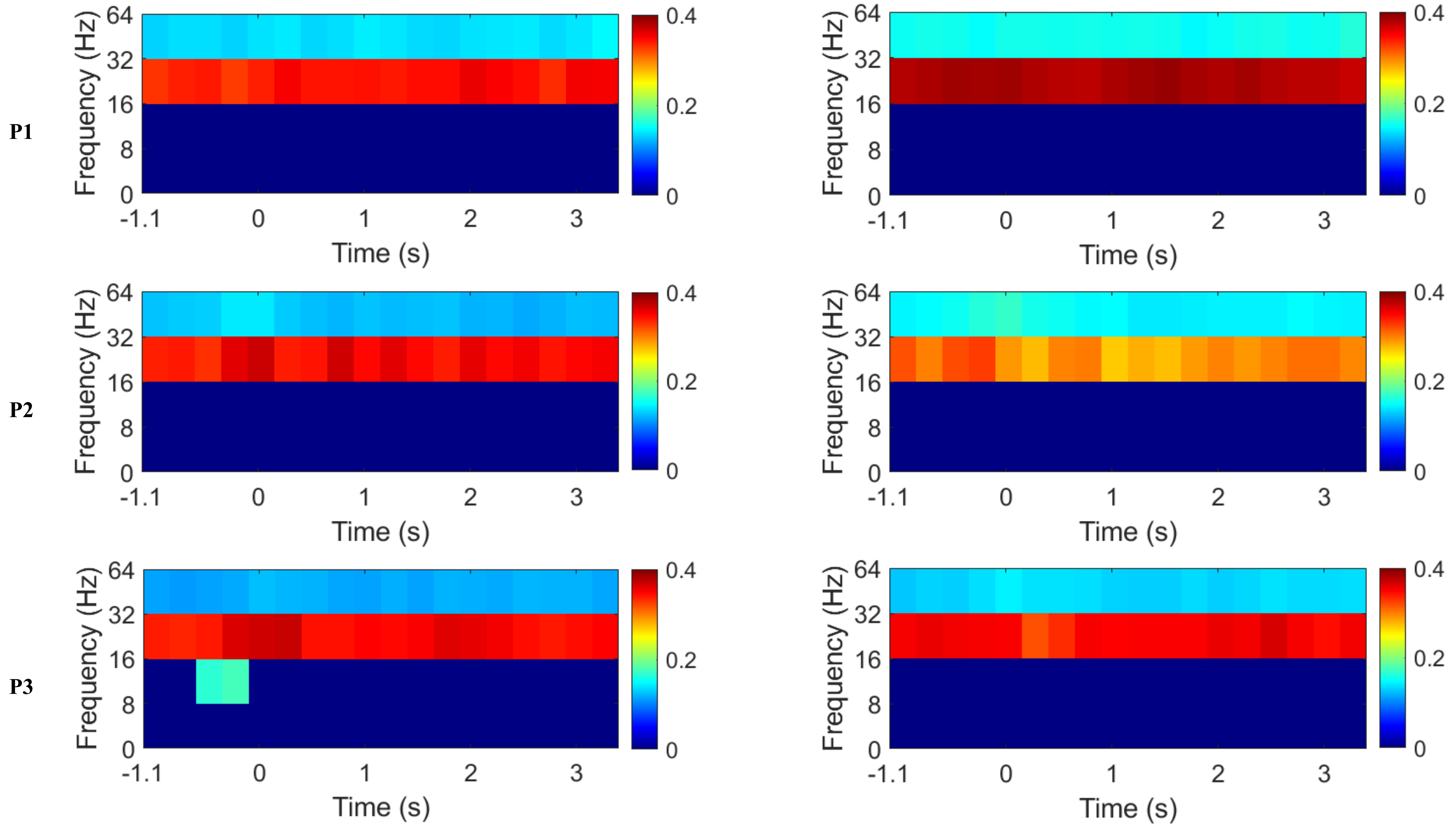
P1
P2
P3
Frequency (Hz)
64
32
16
8
0
Time (s)
-1.1
0
1
2
3
0.4
0.2
0

P4

P5

P6

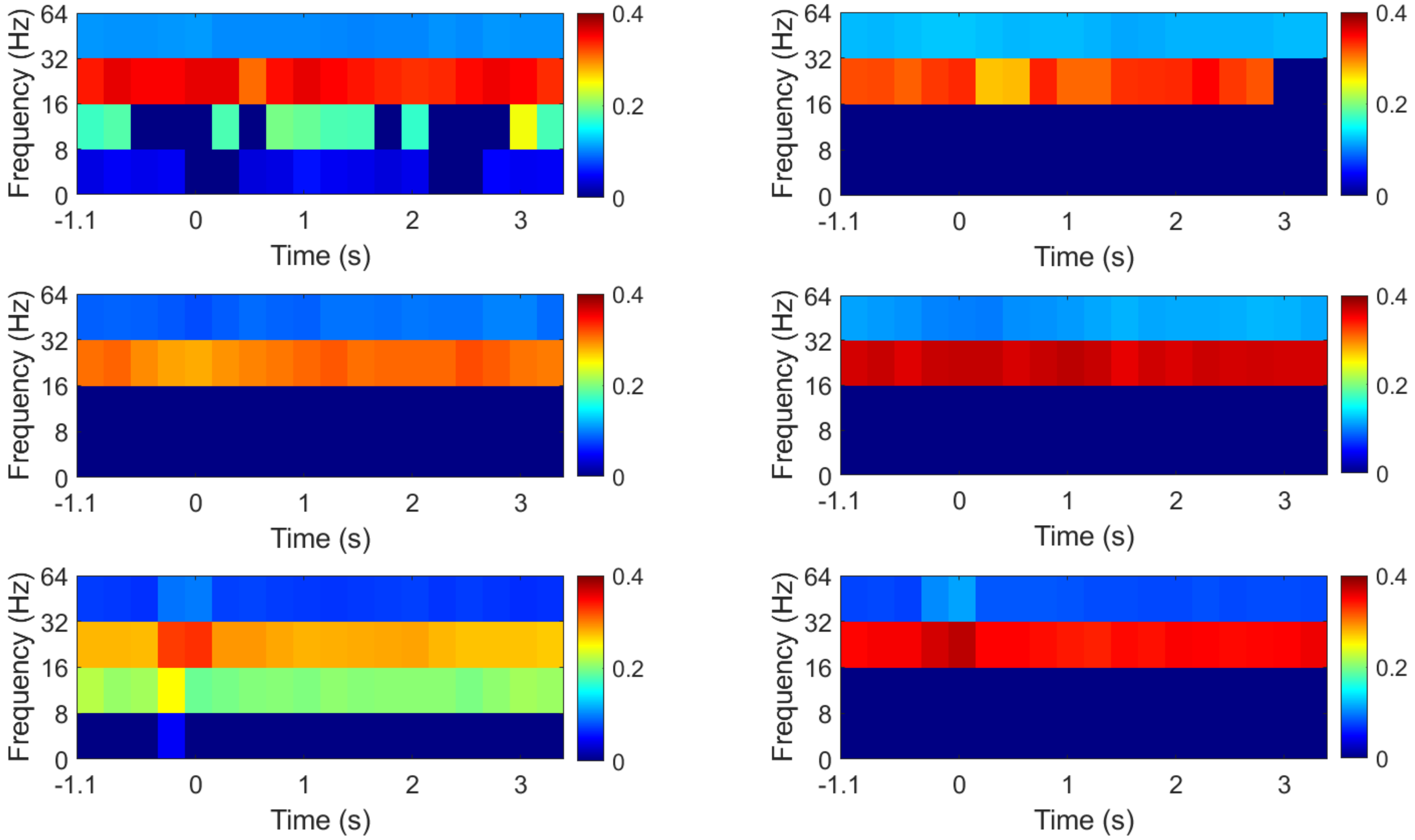

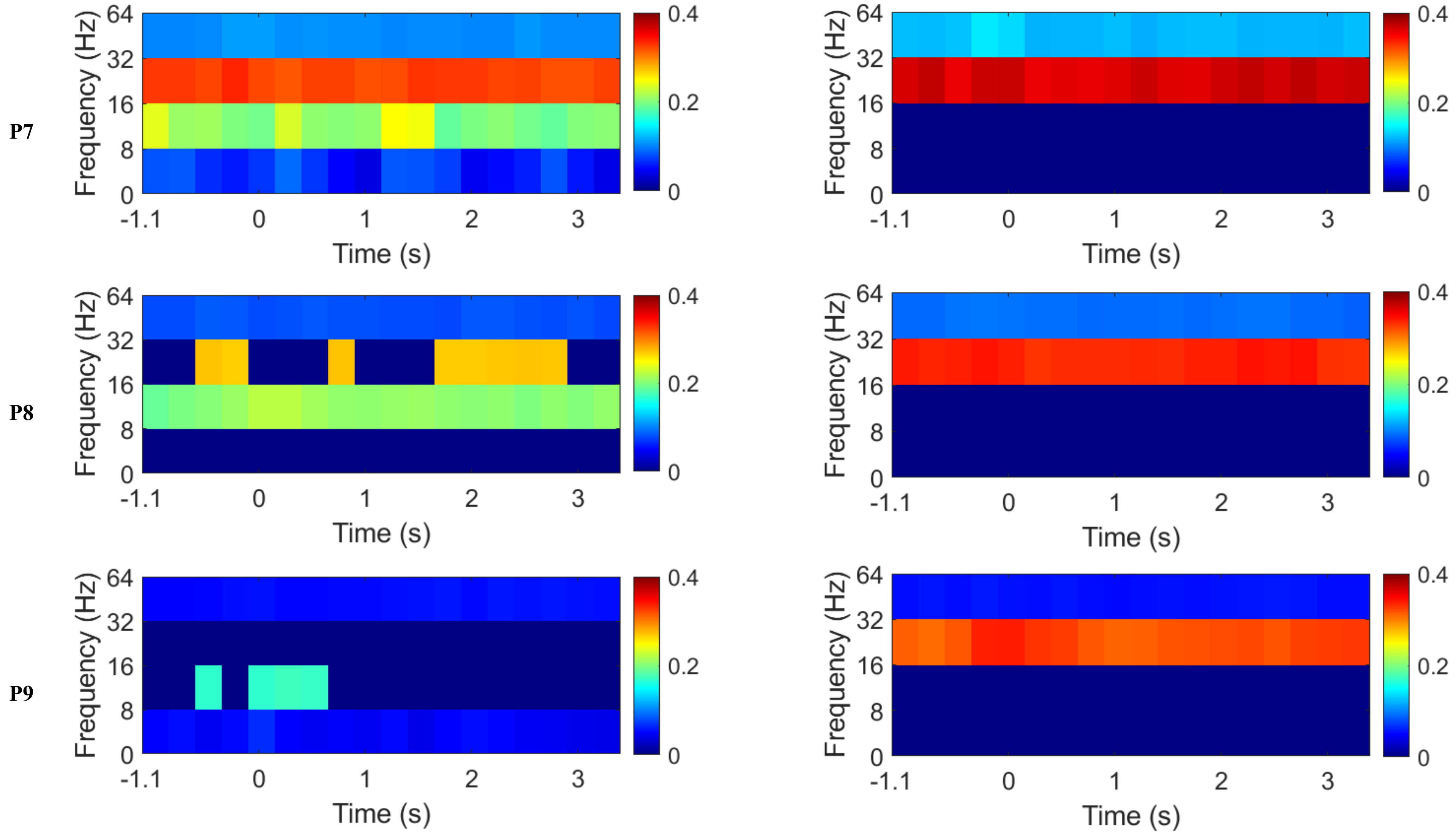
P7
P8
P9
Frequency (Hz)
64
32
16
8
0
Time (s)
-1.1
0
1
2
3
0.4
0.2
0

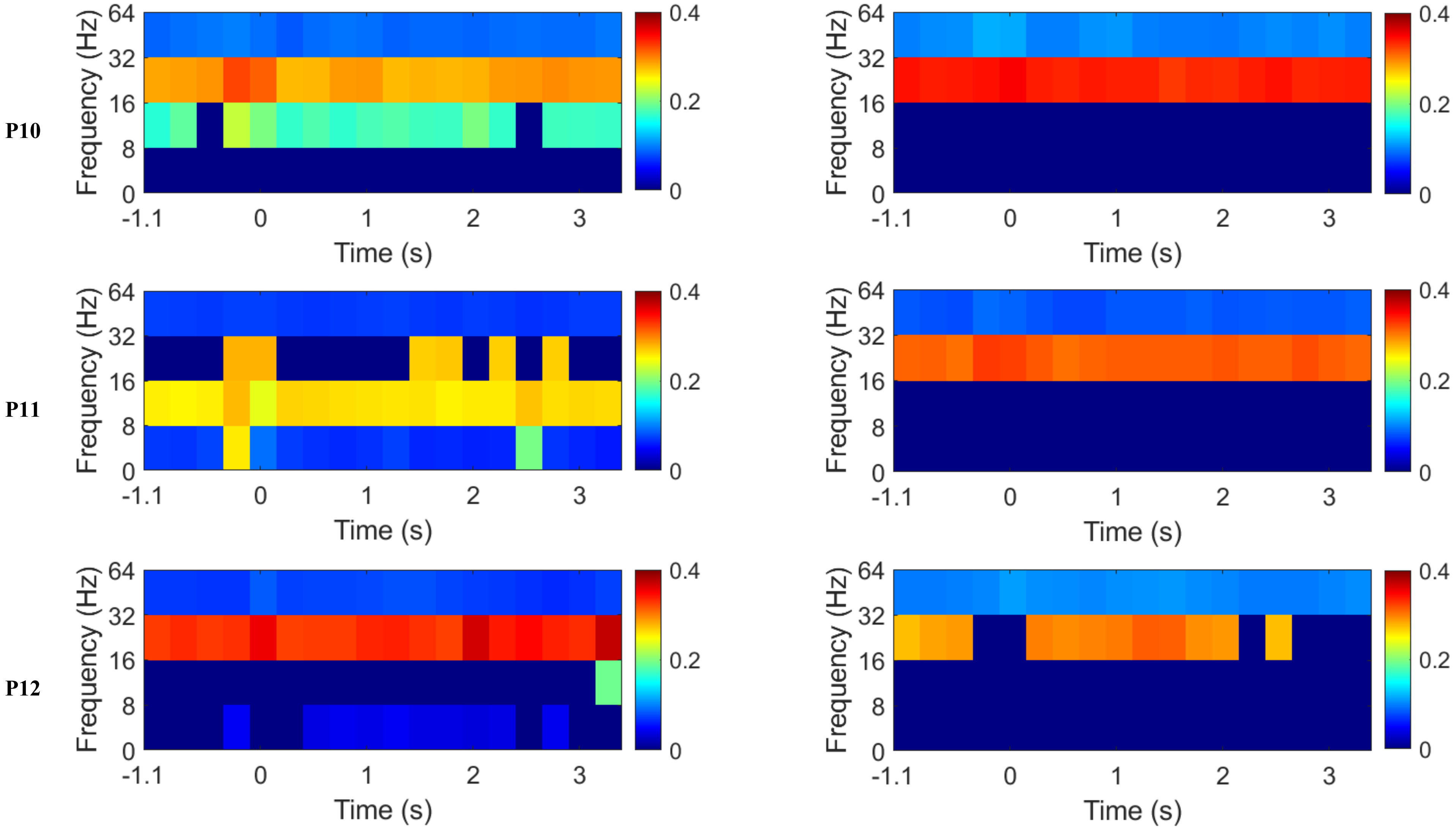
P10
P11
P12
Frequency (Hz)
64
32
16
8
0
Time (s)
-1.1
0
1
2
3
0.4
0.2
0

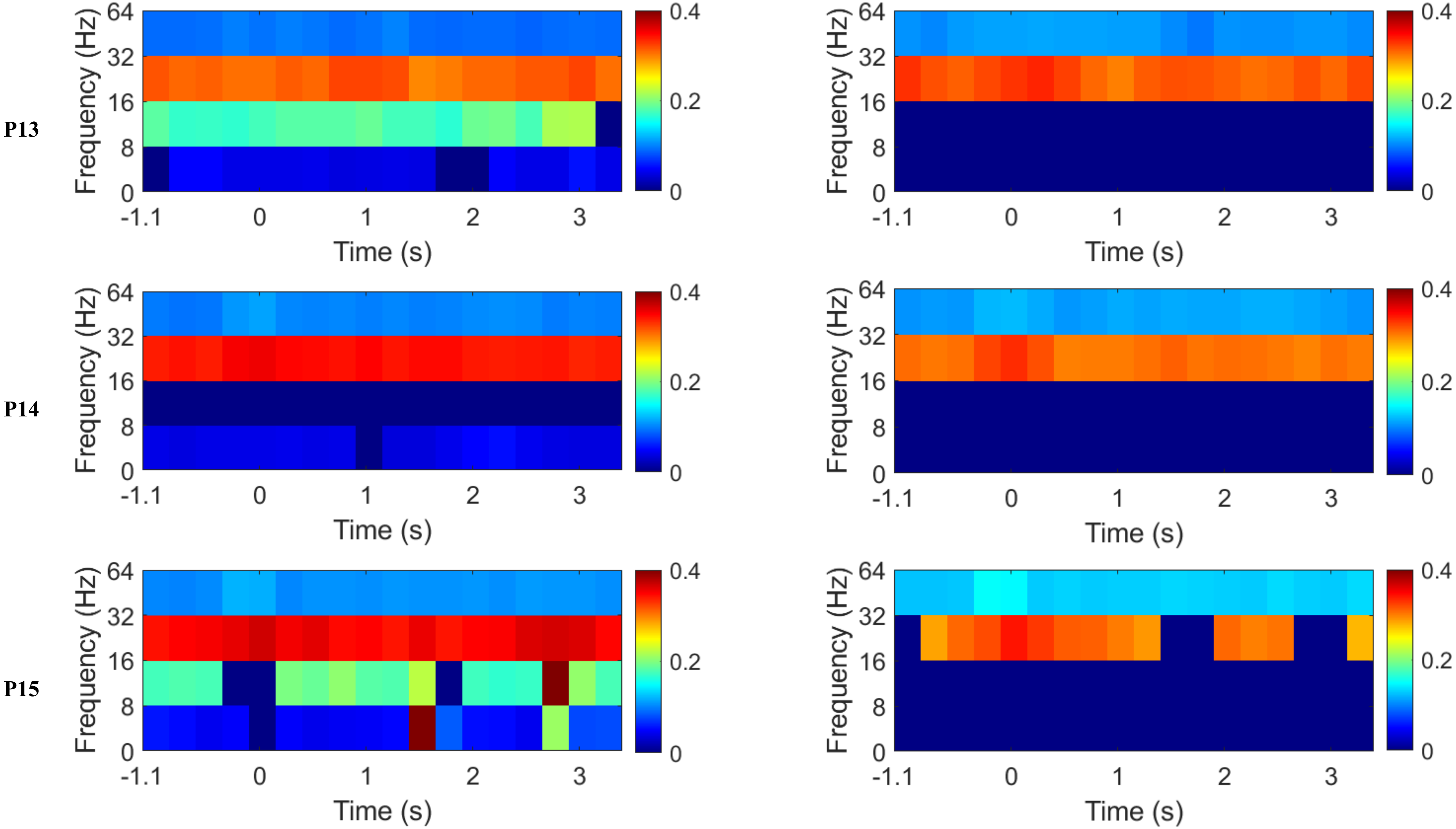
P13
P14
P15
Frequency (Hz)
64
32
16
8
0
-1.1
0
1
2
3
Time (s)
0.4
0.2
0

Supplementary Figure 2 Cross-frequency multi-scale wavelet transfer entropy (MWTE) comparison between control participants and young people with dystonia in the baseline period [-1.1, -0.6] s. Cross-frequency MWTE in 13 control participants (C1-C13) and 15 children with dystonia (P1-P15), focusing on four functional bands, delta/theta (0-8) Hz, alpha (8-16) Hz, beta (16-32) Hz, and low gamma (32-64) Hz. The horizontal axis represents the source, whereas the vertical axis represents the destination. Gaussian white noise signals were employed to generate empirical distributions of cross-frequency MWTE values, mirroring independent processes. The thresholds for significance were determined by the 95th percentiles of the respective empirical distributions, effectively representing approximately 95% confidence intervals. Any values falling below these established thresholds are regarded as non-significant and are consequently assigned a value of zero, visually depicted by the colour dark blue.

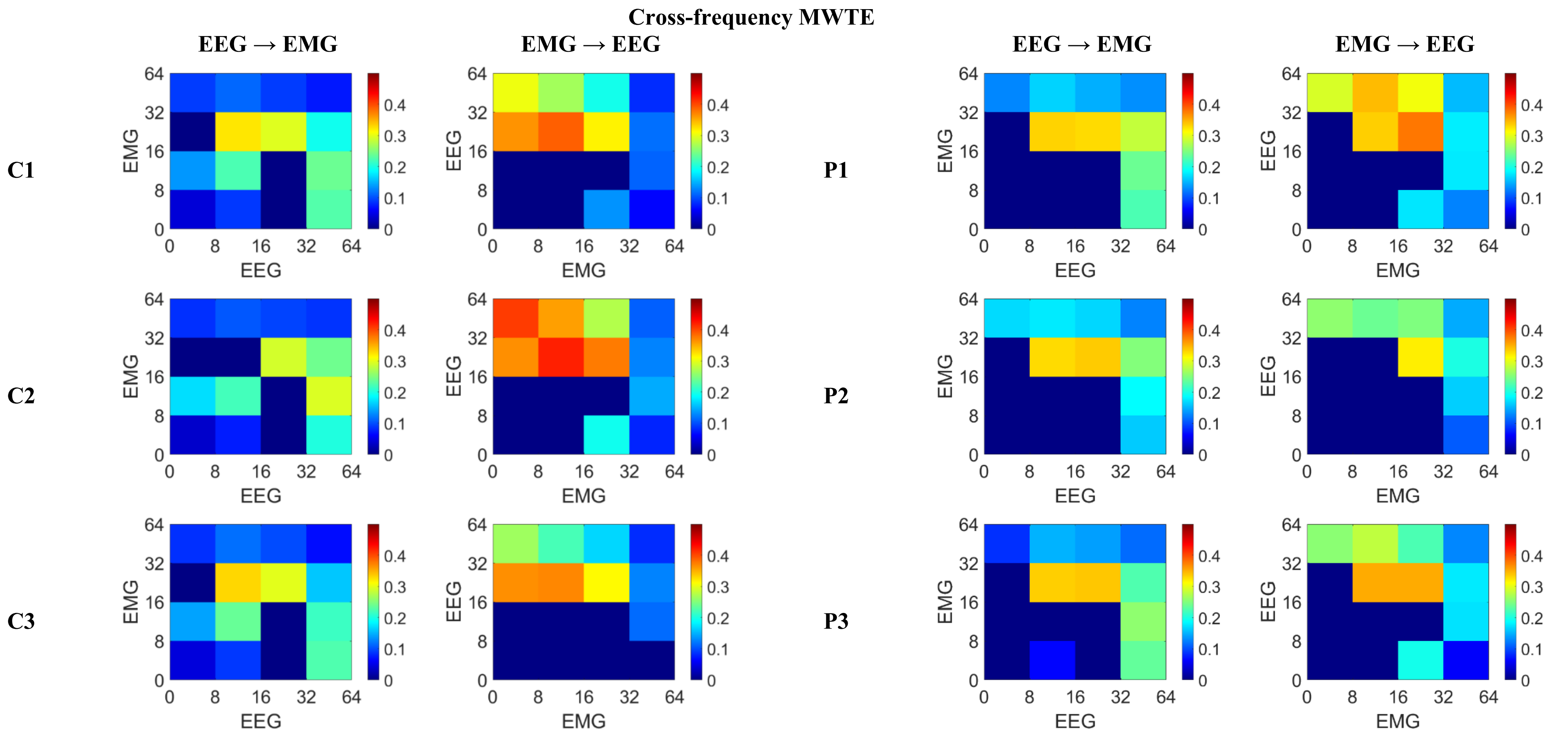

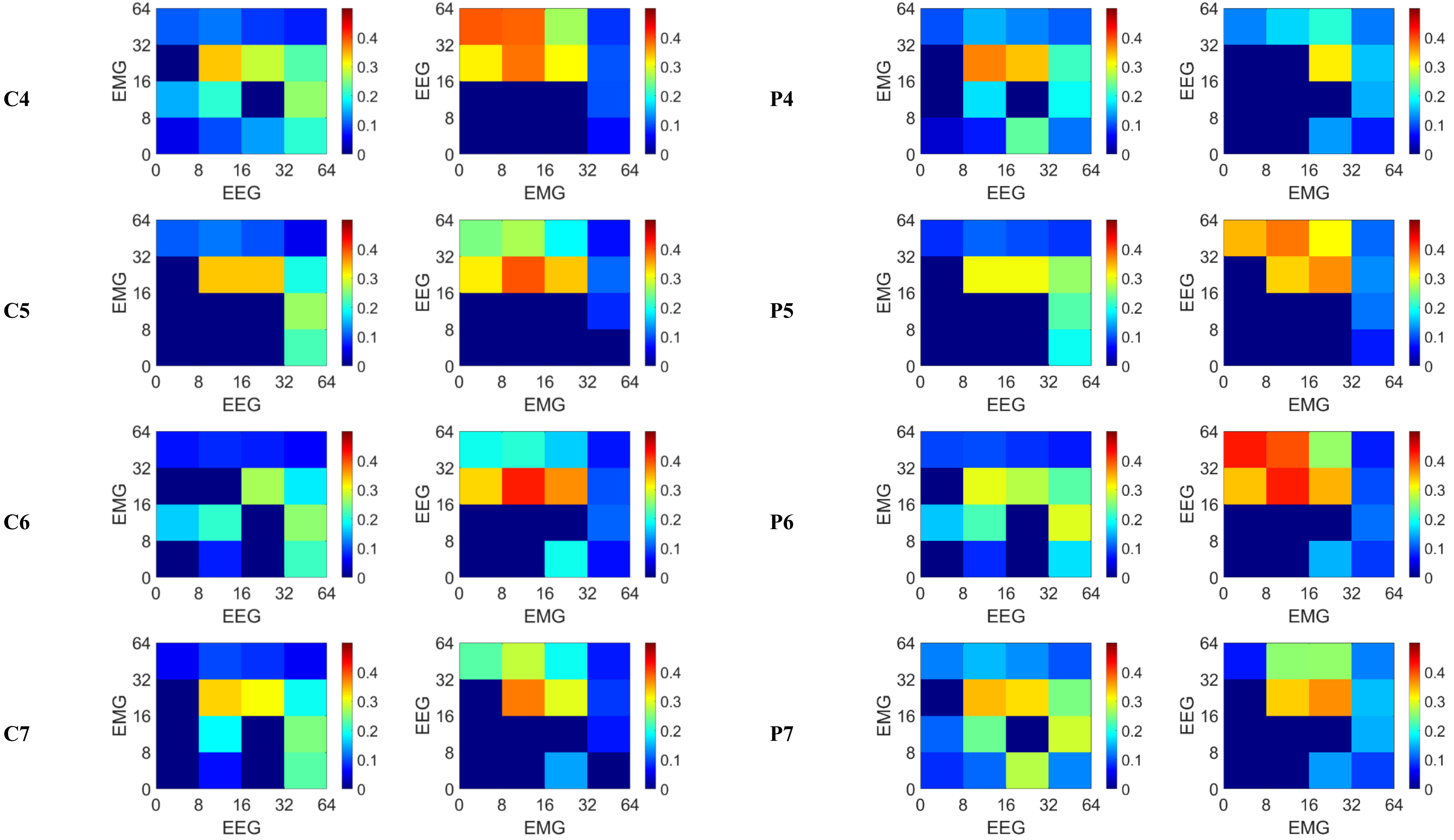

C4
C5
C6
C7
P4
P5
P6
P7
EEG
EMG
0
8
16
32
64
0
0.1
0.2
0.3
0.4

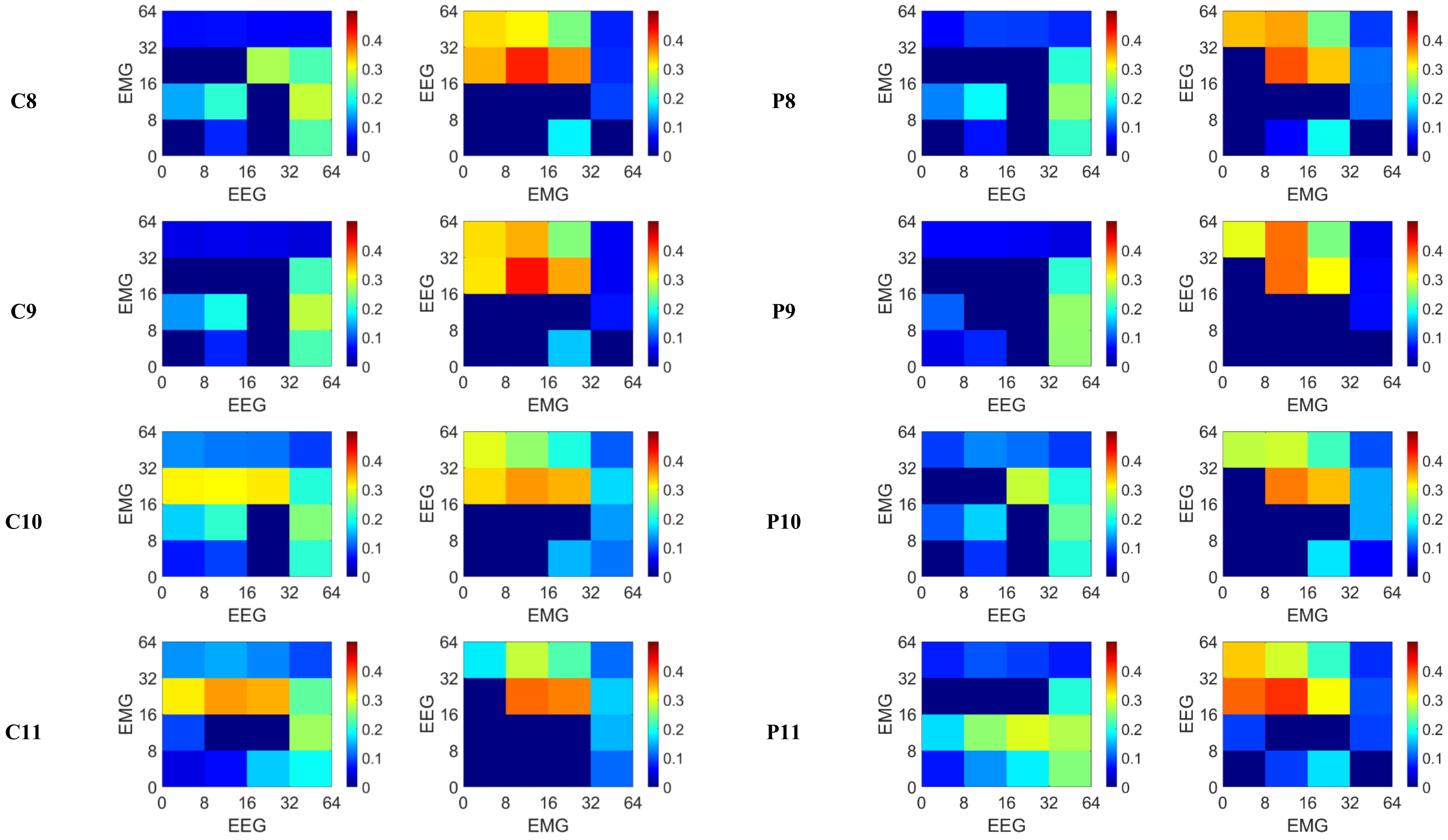
C8
C9
C10
C11
P8
P9
P10
P11
EEG
EMG
0 8 16 32 64
0 0.1 0.2 0.3 0.4

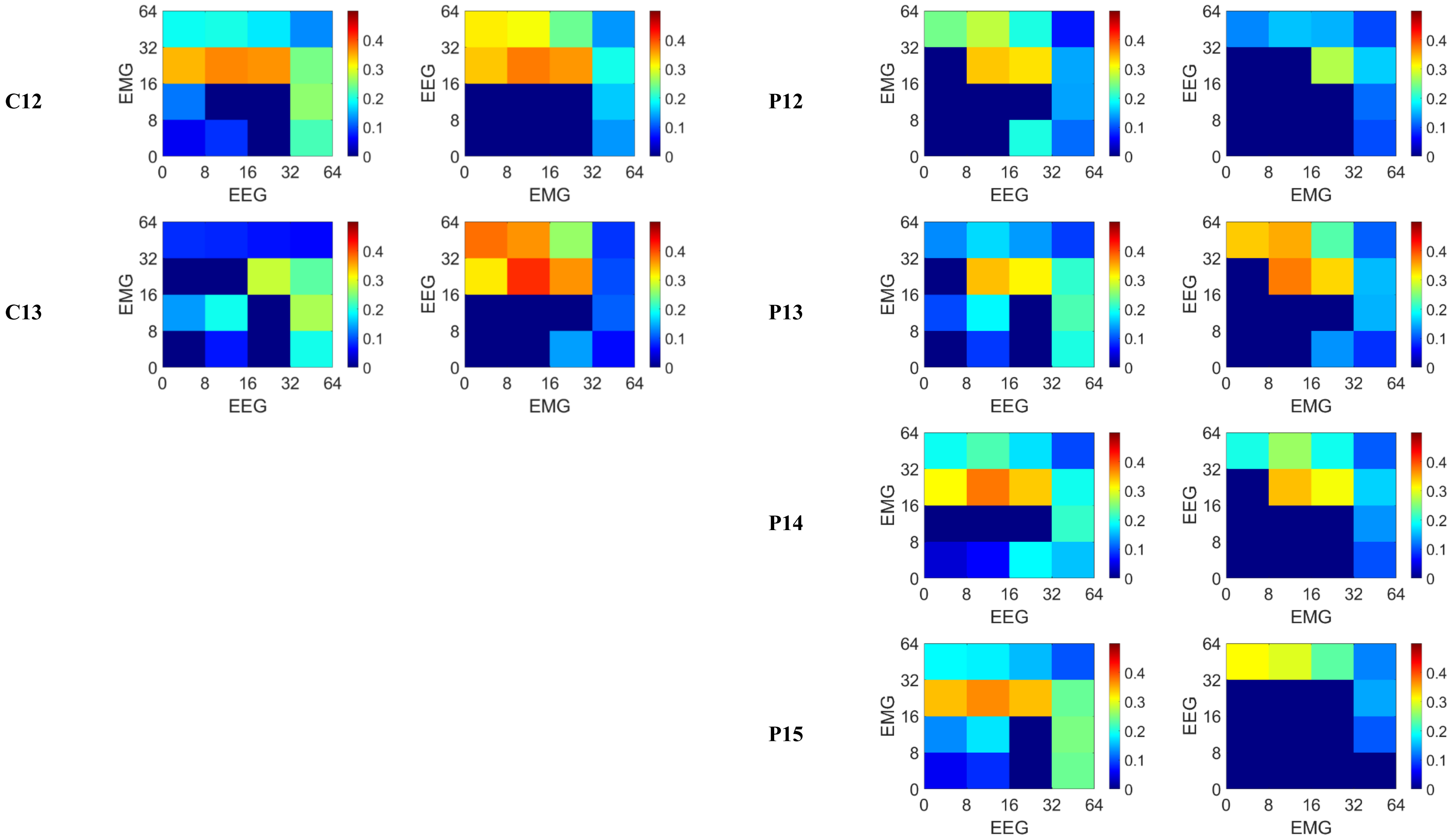

C12
C13
P12
P13
P14
P15
EMG
EEG
0
8
16
32
64
0
0.1
0.2
0.3
0.4

Supplementary Figure 3 Cross-frequency multi-scale wavelet transfer entropy (MWTE) comparison between control participants and young people with dystonia in the early post-stimulus period [0.4, 0.9] s. Cross-frequency MWTE in 13 control participants (C1-C13) and 15 children with dystonia (P1-P15), focusing on four functional bands, delta/theta (0-8) Hz, alpha (8-16) Hz, beta (16-32) Hz, and low gamma (32-64) Hz. The horizontal axis represents the source, whereas the vertical axis represents the destination. Gaussian white noise signals were employed to generate empirical distributions of cross-frequency MWTE values, mirroring independent processes. The thresholds for significance were determined by the 95th percentiles of the respective empirical distributions, effectively representing approximately 95% confidence intervals. Any values falling below these established thresholds are regarded as non-significant and are consequently assigned a value of zero, visually depicted by the colour dark blue.

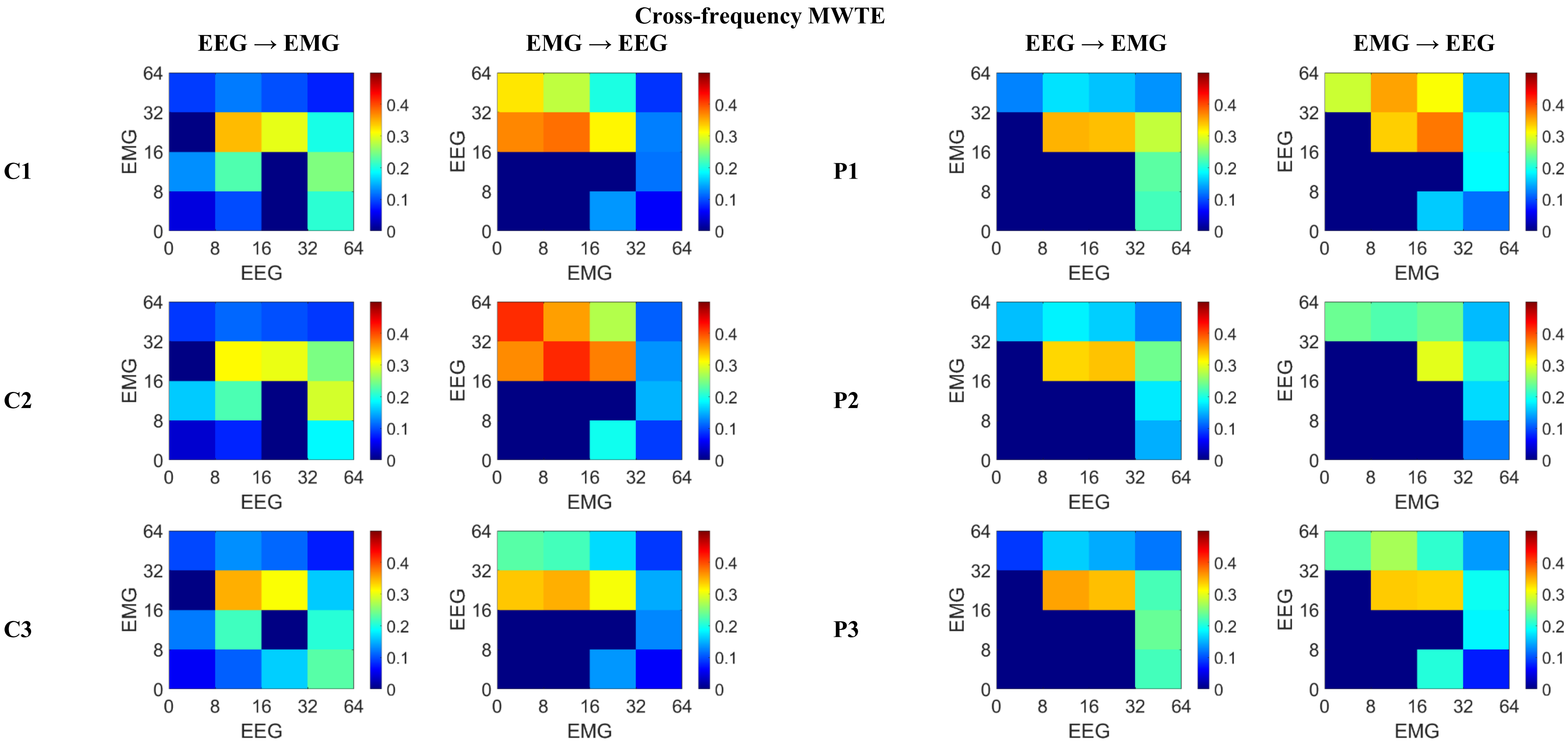

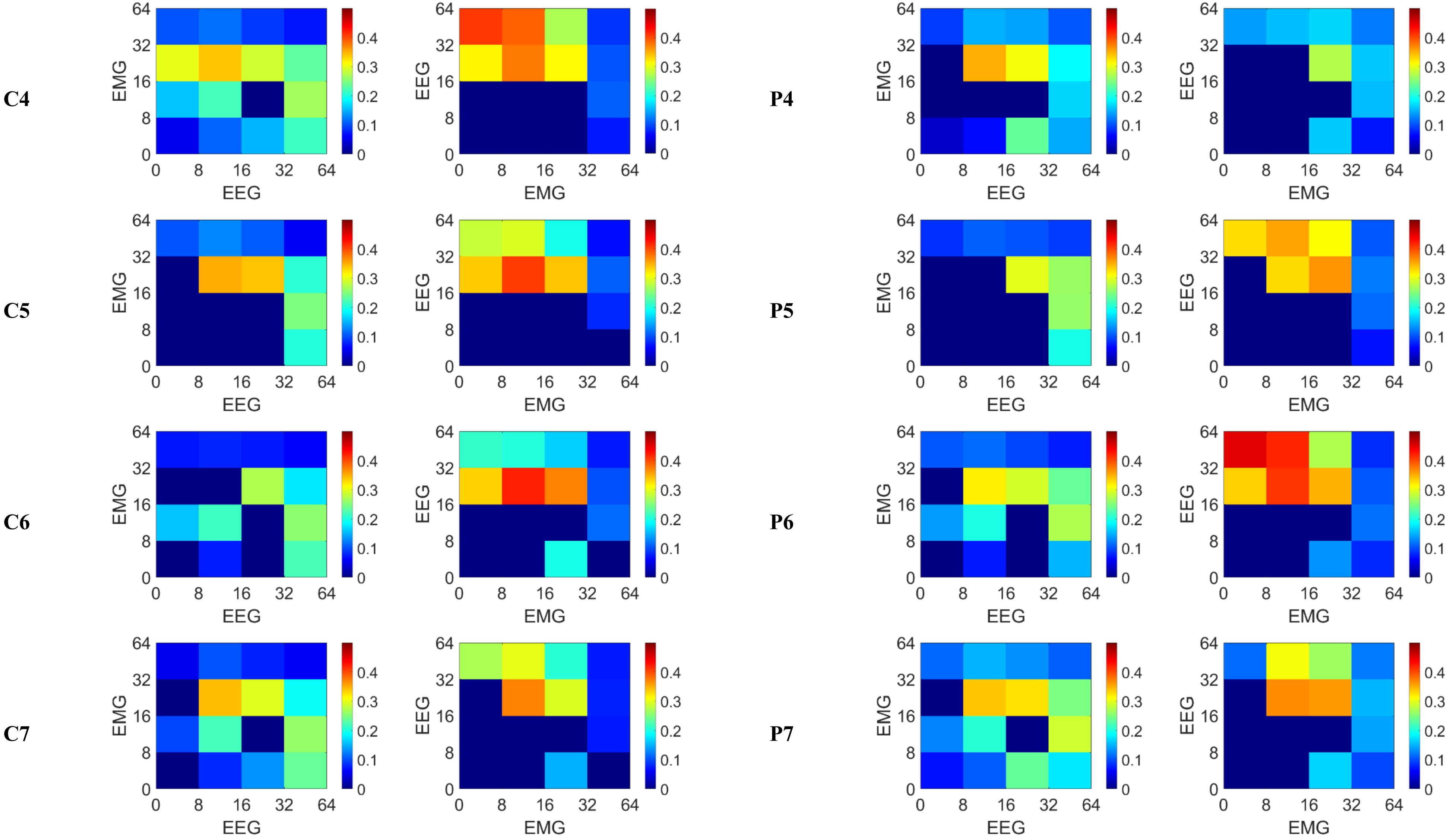
C4
C5
C6
C7
P4
P5
P6
P7
EEG
EMG

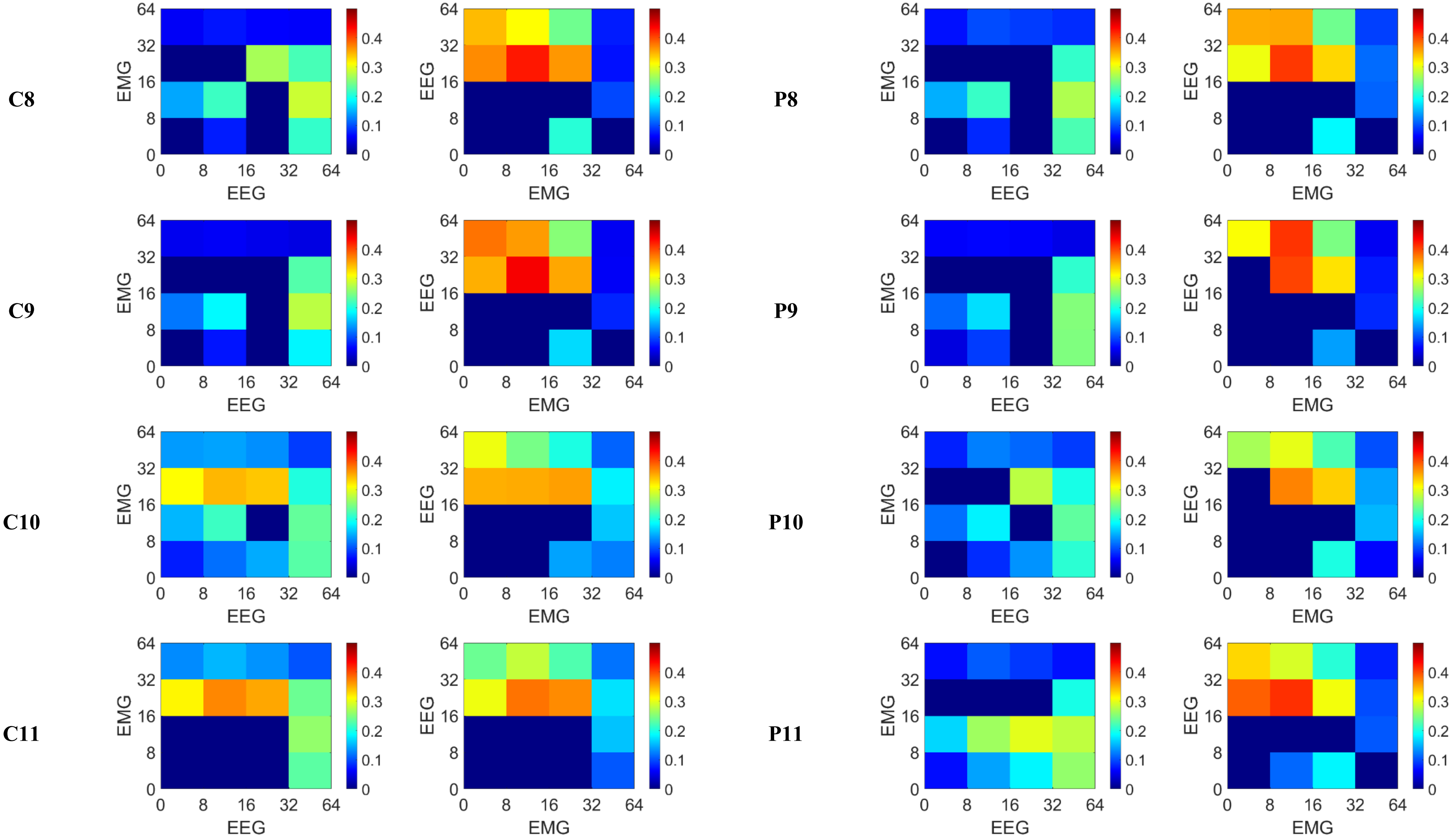
C8
C9
C10
C11
P8
P9
P10
P11
EEG
EMG
0
8
16
32
64
0
0.1
0.2
0.3
0.4

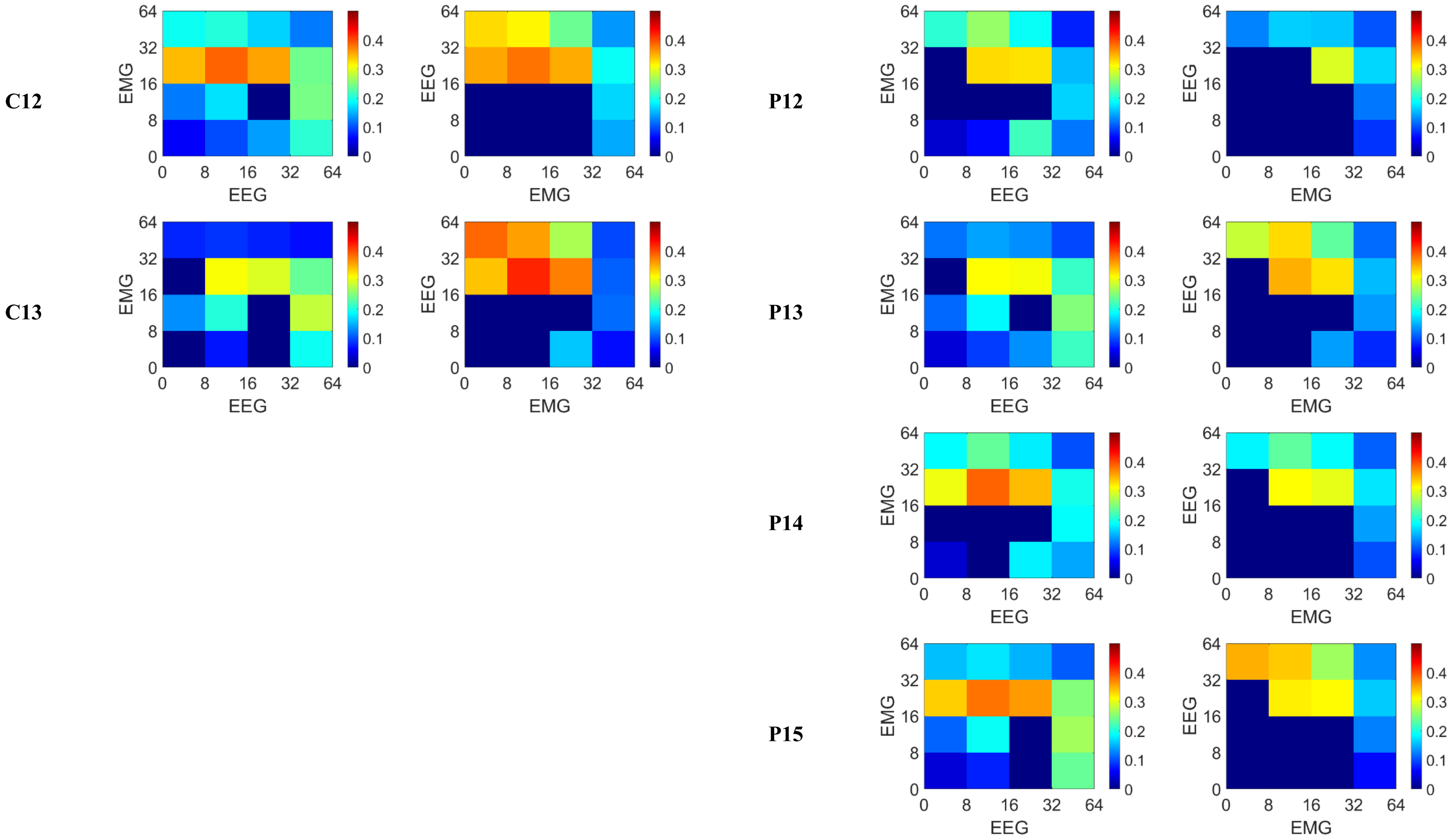
C12
C13
P12
P13
P14
P15
EMG
EEG
0
8
16
32
64
0.4
0.3
0.2
0.1

Supplementary Table 1 Comparison of the proportion of individuals in each group with significant multi-scale wavelet transfer entropy (MWTE) during the early post-stimulus period [0.4, 0.9] s.

EEG (horizontal axis) → EMG (vertical axis)

**Control participants**				
**32-64**	13/13 (100%)	13/13 (100%)	13/13 (100%)	*13/13 (100%)*
**16-32**	4/13 (31%)	10/13 (77%)	*12/13 (92%)*	13/13 (100%)
**8-16**	11/13 (85%)	*11/13 (85%)*	0/13 (0%)	13/13 (100%)
**0-8**	*6/13 (46%)*	11/13 (85%)	5/13 (38%)	13/13 (100%)
**Freq**	**0-8**	**8-16**	**16-32**	**32-64**
**Children with dystonia**				
**32-64**	15/15 (100%)	15/15 (100%)	15/15 (100%)	*15/15 (100%)*
**16-32**	2/15 (13%)	10/15 (67%)	*12/15 (80%)*	15/15 (100%)
**8-16**	8/15 (53%)	*8/15 (53%)*	1/15 (7%)	15/15 (100%)
**0-8**	*8/15 (53%)*	10/15 (67%)	7/15 (47%)	15/15 (100%)
**Freq**	**0-8**	**8-16**	**16-32**	**32-64**
**Fisher's exact test (*p*-value)**				
**32-64**	1.0000	1.0000	1.0000	*1.0000*
**16-32**	0.3720	0.6860	*0.6000*	1.0000
**8-16**	0.1145	*0.1145*	1.0000	1.0000
**0-8**	*1.0000*	0.3955	0.7177	1.0000
**Freq**	**0-8**	**8-16**	**16-32**	**32-64**
**FDR-adjusted *p*-value (*q*-value)**				
**32-64**	1.0000	1.0000	1.0000	*1.0000*
**16-32**	1.0000	1.0000	*1.0000*	1.0000
**8-16**	1.0000	*1.0000*	1.0000	1.0000
**0-8**	*1.0000*	1.0000	1.0000	1.0000
**Freq**	**0-8**	**8-16**	**16-32**	**32-64**

EMG (horizontal axis) → EEG (vertical axis)

**Control participants**				
**32-64**	13/13 (100%)	13/13 (100%)	13/13 (100%)	*13/13 (100%)*
**16-32**	**12/13 (92%)**	13/13 (100%)	*13/13 (100%)*	13/13 (100%)
**8-16**	0/13 (0%)	*0/13 (0%)*	0/13 (0%)	13/13 (100%)
**0-8**	*0/13 (0%)*	0/13 (0%)	9/13 (69%)	8/13 (62%)
**Freq**	**0-8**	**8-16**	**16-32**	**32-64**
**Children with dystonia**				
**32-64**	15/15 (100%)	15/15 (100%)	15/15 (100%)	*15/15 (100%)*
**16-32**	**3/15 (20%)**	12/15 (80%)	*15/15 (100%)*	15/15 (100%)
**8-16**	0/15 (0%)	*0/15 (0%)*	0/15 (0%)	15/15 (100%)
**0-8**	*0/15 (0%)*	1/15 (7%)	10/15 (67%)	12/15 (80%)
**Freq**	**0-8**	**8-16**	**16-32**	**32-64**
**Fisher's exact test (*p*-value)**				
**32-64**	1.0000	1.0000	1.0000	*1.0000*
**16-32**	**0.0002**	0.2262	*1.0000*	1.0000
**8-16**	1.0000	*1.0000*	1.0000	1.0000
**0-8**	*1.0000*	1.0000	1.0000	0.4097
**Freq**	**0-8**	**8-16**	**16-32**	**32-64**
**FDR-adjusted *p*-value (*q*-value)**				
**32-64**	0.9723	0.9723	0.9723	*0.9723*
**16-32**	**0.0031**	0.9723	*0.9723*	0.9723
**8-16**	0.9723	*0.9723*	0.9723	0.9723
**0-8**	*0.9723*	0.9723	0.9723	0.9723
**Freq**	**0-8**	**8-16**	**16-32**	**32-64**

Summary of number of individuals showing significant cross-frequency coupling (CFC), in either direction: EEG → EMG (left table) and EMG → EEG (right table). For each sub-table, the horizontal frequency axis represents the source, whereas the vertical axis represents the destination. The intra-frequency coupling combinations are shown in italics. A Fisher's exact test was applied to compare the proportions of individuals in each group showing significant coupling for each CFC combination. Storey's FDR method was used to account for multiple comparisons, and the hypothesis testing error measures (*q* values) were adjusted accordingly. Highlighted in bold: $q < 0.05$.

Supplementary Table 2 Comparison of the mean level of multi-scale wavelet transfer entropy (MWTE) between groups during the early post-stimulus period [0.4, 0.9] s.

EEG (horizontal axis) → EMG (vertical axis)

**Two-sample *t*-test (*p*-value)**				
**32-64**	0.2926	0.0673	0.0282	*0.0233*
**16-32**	0.1236	0.5362	*0.8246*	0.5959
**8-16**	0.0269	*0.0368*	0.2000	0.0980
**0-8**	*0.4811*	0.2397	0.2477	0.1881
**Freq**	**0-8**	**8-16**	**16-32**	**32-64**
**FDR-adjusted *p*-value (*q*-value)**				
**32-64**	0.3459	0.2189	0.1469	*0.1469*
**16-32**	0.2222	0.5363	*0.6918*	0.5740
**8-16**	0.1469	*0.1593*	0.2891	0.2222
**0-8**	*0.5213*	0.3133	0.3133	0.2877
**Freq**	**0-8**	**8-16**	**16-32**	**32-64**
**Two-sample effect size (Cohen's *d*)**				
**32-64**	-0.3952	-0.7023	-0.8547	*-0.8870*
**16-32**	0.5855	0.2306	*-0.0824*	-0.1975
**8-16**	0.8632	*0.8096*	0.4837	0.6313
**0-8**	*0.2630*	0.4427	-0.4350	0.4973
**Freq**	**0-8**	**8-16**	**16-32**	**32-64**

EMG (horizontal axis) → EEG (vertical axis)

**Two-sample *t*-test (*p*-value)**				
**32-64**	0.1177	0.9790	0.4540	*0.0429*
**16-32**	**0.0001**	0.0061	*0.0796*	0.0943
**8-16**	0.1252	*0.7396*	0.1281	0.1777
**0-8**	*0.7433*	0.2530	0.5143	0.8188
**Freq**	**0-8**	**8-16**	**16-32**	**32-64**
**FDR-adjusted *p*-value (*q*-value)**				
**32-64**	0.2222	0.7957	0.5134	*0.1593*
**16-32**	**0.0023**	0.0799	*0.2222*	0.2222
**8-16**	0.2222	*0.6666*	0.2222	0.2877
**0-8**	*0.6666*	0.3133	0.5351	0.6918
**Freq**	**0-8**	**8-16**	**16-32**	**32-64**
**Two-sample effect size (Cohen's *d*)**				
**32-64**	0.5952	0.0098	-0.2796	*-0.7833*
**16-32**	**1.7023**	1.0971	*0.6711*	-0.6389
**8-16**	0.5829	*0.1236*	0.5781	-0.5096
**0-8**	*-0.1218*	-0.4301	0.2432	-0.0851
**Freq**	**0-8**	**8-16**	**16-32**	**32-64**

Comparison of the mean level of MWTE in the control group and the mean value of MWTE in the dystonia group, in either direction: EEG → EMG (left table) and EMG → EEG (right table). For each sub-table, the horizontal frequency axis represents the source, whereas the vertical axis represents the destination. The intra-frequency coupling combinations are shown in italics. A two-sample *t*-test is applied to investigate whether the unknown population means of two groups are statistically equal or not. Storey's FDR method was used to account for multiple comparisons, and the hypothesis testing error measures (*q* values) were adjusted accordingly. Cohen's *d* was employed as a measure of effect size to accompany reporting of two-sample *t*-test results. Highlighted in bold: $q < 0.05$.

Supplementary Table 3 Linear regression analysis for the consideration of confounding factors.

	**Time Window**	**[-1.1, -0.6] s**	**[-0.6, -0.1] s**	**[-0.1, 0.4] s**	**[0.4, 0.9] s**	**[0.9, 1.4] s**	**[1.4, 1.9] s**	**[1.9, 2.4] s**	**[2.4, 2.9] s**	**[2.9, 3.4] s**
**Group Assignment**	***t*-statistic**	**-2.6459**	**-2.0753**	**-4.6943**	**-3.1982**	-1.2638	**-2.5361**	-1.3082	**-2.2679**	**-3.2577**
	p	**0.0142**	**0.0488**	**0.0001**	**0.0039**	0.2184	**0.0181**	0.2032	**0.0326**	**0.0033**
**% MVC**	***t*-statistic**	0.0836	-0.9300	0.3151	-0.5015	-1.5526	0.1202	-1.4083	-0.2690	0.3072
	p	0.9341	0.3616	0.7554	0.6206	0.1336	0.9053	0.1719	0.7903	0.7613
**% CV**	***t*-statistic**	0.4362	0.4798	-0.6895	0.0437	-1.0156	-0.2598	-1.7347	-0.7060	-0.5745
	p	0.6666	0.6357	0.4971	0.9655	0.3199	0.7972	0.0956	0.4870	0.5710

Linear regression analysis to interpret the model statistics, in which group assignment, level of EMG (expressed as % MVC), and contraction variability (expressed as CV of rectified EMG) are used simultaneously as regressors for multi-scale wavelet transfer entropy (MWTE) from the EMG (0-8 Hz) range to the EEG (16-32 Hz) range in each of the nine non-overlapping time windows across the epoch. The *t*-statistic and the corresponding *p*-value for each of the regressors are displayed. Highlighted in bold: $p < 0.05$.
